# Transfrontier Conservation Areas and Human-Wildlife Conflict: The Case of the Namibian Component of the Kavango-Zambezi (KAZA) TFCA

**DOI:** 10.1038/s41598-020-64537-9

**Published:** 2020-05-14

**Authors:** Mirja Stoldt, Thomas Göttert, Carsten Mann, Ulrich Zeller

**Affiliations:** 1 Namibia Nature Foundation, Dr Frans Indongo Street 76 & 78, Windhoek, Namibia; 2 Systematic Zoology Division, Albrecht Daniel Thaer-Institute of Agricultural and Horticultural Sciences, Faculty of Life Sciences, Humboldt-Universität zu Berlin, Unter den Linden 6, 10099 Berlin, Germany; 3 Eberswalde University for Sustainable Development, Department of Sustainable Forest Resource Economics, Alfred-Möller-Str. 1, 16225 Eberswalde, Germany; 4 Systematic Zoology Division, Albrecht Daniel Thaer-Institute of Agricultural and Horticultural Sciences, Faculty of Life Sciences, Humboldt-Universität zu Berlin, Unter den Linden 6, 10099 Berlin, Germany

**Keywords:** Animal migration, Biodiversity, Conservation biology

## Abstract

Our study deals with human-wildlife conflicts in the Namibian component of the Kavango-Zambezi Transfrontier Conservation Area (KAZA TFCA). The study reconstructs the historical occurrence of selected mammal species and adopts a socio-ecological approach to assess the impact of human dimensions in the KAZA TFCA. Our results reveal pronounced human–wildlife conflicts with considerable impacts on the livelihoods of communities. Human–wildlife conflict has the potential to become a significant contributor to the failure of the TFCA concept. Conflicts are influenced by a growing human population and large mammal species re-colonising formerly abandoned areas. Mapping the occurrence of selected mammal species over time reveals an interesting picture: although conservation initiatives have led to an increase in the population size of selected species, their occurrence is more restricted than in times of heavily decimated wildlife populations. The increasing restriction of wildlife to protected areas reduces the resilience of the ecosystem. To sustainably manage and conserve wildlife populations, a bigger picture including areas outside of the current borders of KAZA TFCA should be considered. This could support re-connecting ecologically important areas for congested populations to move to and reduces the concentration of wildlife and pressure on the land and people of the region.

## Introduction

The proclamation and development of Transfrontier Parks (TFP) and Transfrontier Conservation Areas (TFCA) according to the Peace Parks concept is based on a fundamental re-thinking of protected area concepts and management. It marks an important milestone in nature conservation, poverty reduction and securing peace in Sub-Saharan Africa^1^. In contrast to the colonial “fortress” or “fences-and-fines” approach to conservation, that alienated society from nature^2^, current strategies focus on managing entire bioregions based on the sustainable utilisation of biodiversity and involving multiple stakeholders^1,3^. Transfrontier Parks (TFP), thus, acknowledge the importance of ecological, physio-geographic and socio-cultural, anthropogenic factors that influence an area^4^.

The Kavango-Zambezi Transfrontier Conservation Area (KAZA TFCA) was established in 2011 by its member states Angola, Botswana, Namibia, Zambia and Zimbabwe. This conservation area is considered to be an important means to create economic development and conserve the unique biodiversity within the region with particular focus on large-scale migrations of megafauna. One of the main challenges the KAZA TFCA is facing is how to control human-wildlife conflict and accommodate growing human populations, increasing wildlife populations and linking protected areas.

The KAZA TFCA aims to create free movement for wildlife and increase wildlife dispersal areas to target these challenges and counteract the trend of fortress conservation. Creating free movement for wildlife opens up areas for congested populations, enlarging the effective distribution range, supporting meta-population management and thus, increasing ecological stability^5^. It could, however, also lead to some areas becoming a “transit-route” for wildlife thereby increasing human–wildlife conflict due to increasing human wildlife encounters/interactions. This creates a human-wildlife conflict dilemma: larger areas created to reduce human-wildlife conflict tend to accelerate conflict as wildlife populations recover. Namibia has implemented exemplary conservation strategies, setting aside large areas for conservation and involving local communities in conservation, thereby increasing the populations of many species throughout the country. To control human-wildlife conflict new management strategies, that could be of considerable importance to the rest of the region and the world^6^, need to be implemented.

According to Madden^7^, [p. 248], human-wildlife conflict (HWC) occurs “*when the needs and behaviour of wildlife impact negatively on the goals of humans or when the goals of humans negatively impact the needs of wildlife. These conflicts may result when wildlife damage crops, injure or kill domestic animals, threaten or kill people*.” Important global drivers of HWC are human population growth and land-use transformation. As humans encroach into wildlife habitat and transform these areas into settlements or agrarian areas, wildlife habitat is lost, fragmented or degraded. Increasing livestock populations compete for grazing and foraging with wild herbivores leading to declines in their populations. Research shows, that predators prefer wild prey when they are abundant. Declining wild prey populations can cause predators to shift their diet to livestock. Human-wildlife conflict is also driven by increasing wildlife populations due to effective conservations efforts. These increasing populations cannot be confined to human defined ranges. In addition, the increasing interest in ecotourism leads to an increasing human presence in protected areas raising concerns about the management of these areas. Human-wildlife conflict can also be indirectly influenced by climatic events, which can influence hunting strategies, or stochastic events (e.g. fire), which can cause animals to flee towards human settlements^8^.

One key aim of the KAZA TFCA is to connect and coordinate efforts of protected areas within its borders. This involves creating common ground among five states with different legislation, land-uses, attitudes to conservation and at different stages of development. This creates a very complex situation. To look at a more manageable unit of analysis in detail we will focus on the Namibian component of the KAZA TFCA. Namibia has been at the forefront of effective wildlife management and the sustainable use of natural resources in southern Africa and is a good starting point to analyse wildlife movement and management.

This study aims to explore the impacts of different key ecological and socio-economic factors on human-wildlife conflict by adopting a historical perspective. Relevant factors were identified by conducting a literature review and complemented by factors identified during expert interviews and surveys with community members living in the Namibian component of the KAZA TFCA. Changes in the predominant land use, wildlife dispersal areas and migration routes, wildlife numbers, population density and poverty indices are analysed over time to determine relationships and examine patterns of human-wildlife conflict in the Namibian component of the KAZA TFCA. The study aims to answer the question if and how the KAZA TFCA can influence human-wildlife conflict in the Namibian component of the KAZA TFCA. As our guiding hypothesis, we assume that (1) the KAZA TFCA can increase human-wildlife conflict in Namibia by opening up areas for congested wildlife populations to move to, thereby increasing the movement of wildlife through densely populated areas with a high number of poor subsistence farmers (Hypothesis 1) and (2) that historical events can lead to changes in the occurrence and number of species which increased wildlife density in the Namibian component of the KAZA TFCA (Hypothesis 2).

## Study Area

The KAZA TFCA is the largest transfrontier conservation area in the world covering almost 520 000 km^2^ in Angola, Botswana, Zambia, Zimbabwe and Namibia. Of this area, 371 394 km^2^ are under conservation. The remaining 148 520 km^2^ are mainly used for agricultural activities including rangeland^9^. The project is co-funded by the German Kreditanstalt für Wiederaufbau (KfW) and supported by international organisations such as Conservation International and the Peace Parks Foundation^10^. The treaty was signed on the 18 August 2011^8^. The TFCA should contribute to peace and stability within the region, establish links between fragmented habitats and create economic development through tourism^11^. The area hosts the largest elephant population in the world and about 25% of the global wild dog population^12^. According to Fynn and Bonyongo^5^, some parts of the KAZA TFCA belong to the last functional conservation areas in Africa, that include all functional resource gradients required for the seasonal large-scale migration of ungulate species between habitats^5^.

**Figure 1:**
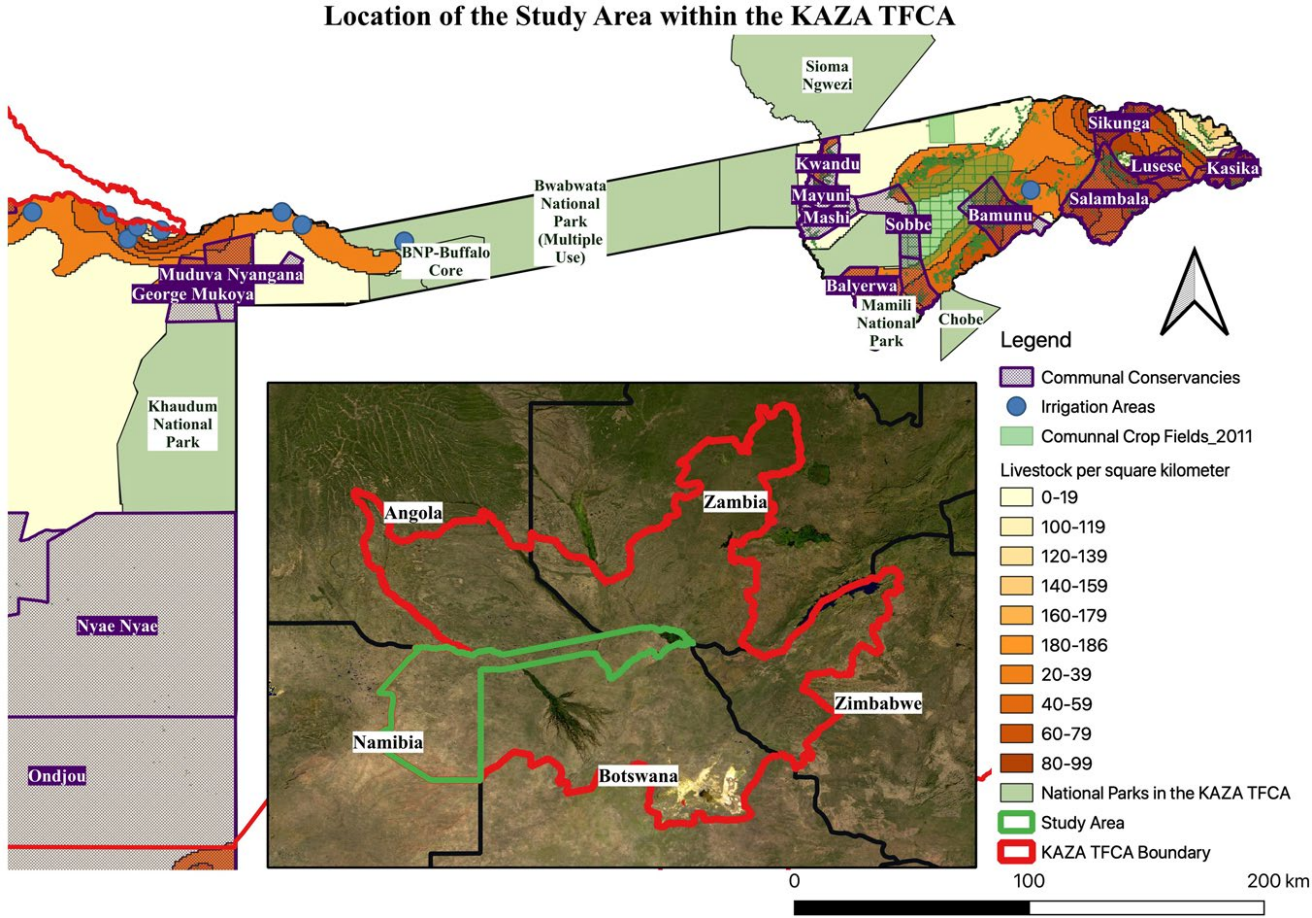
Map of the Namibian component of the Kavango-Zambezi Conservation Area (KAZA TFCA). The map shows the study area including the location of national parks (light green) and conservancies (purple grid), larger irrigation areas (blue points) in the area, areas under crop cultivation (green grid) and livestock densities (various orange shades with the darkest shades having the highest densities) mapped using QGIS 3.12^14^. For orientation purposes it also includes the five KAZA TFCA signatory countries, the location of the KAZA TFCA (red outline) and the study area (green outline).

In this paper, we focus on the Namibian component of the KAZA TFCA as a good example for sustainable protected area management. Namibia has been successful in constitutionalising nature conservation and the sustainable use of natural resources. Some protected areas in the country have existed for more than 110 years. The north-east parks are relatively young but are important refuges for wetland-associated faunal elements and a diverse megafauna, including large aggregated wildlife migrations. They constitute 16% of the Namibian portion of the KAZA TFCA^13^ and include Mangetti, Khaudom, Bwabwata, Mudumu and Nkasa Rupara National Parks (Fig. 1).

The Bwabwata National Park is the largest of these national parks^13^ and is largely fenced except for 30 km east of the Kwando river. It borders the Mashi, Mayuni and Kwando conservancies registered under Namibia’s community-based natural resource management (CBNRM) programme. People are permitted to live within the park, which is divided into multiple-use and core conservation areas^13^. The permission to use natural resources in the multiple-use areas is based on the assumption that people only see the value of wildlife if they benefit from living in harmony with wild animals^14^. The Buffalo Core Area of the Bwabwata National Park hosts a great diversity of large mammals while the Mahango Core Area is recognised as an internationally important bird area^13^. The Buffalo and Kwando Core Areas are also a very important water source for elephants during breeding times^13^. The Mudumu National Park has a key function as a core wildlife area with animals migrating from the park to surrounding conservancies, where they can be used consumptively through quota hunting or non-consumptively through tourism^13^. The Mamili/Nkasa Rupara National Park supports the largest wetland area within Namibia and is made up of channels, lagoons and islands^13^. The Khaudom National Park is fenced along the border with Botswana and unfenced and surrounded by conservancies on the Namibian side. The park is an important migration route for many species travelling towards the Okavango Delta. It is one of the few areas on the African continent, where endangered large mammal species such as the African wild dog (*Lycaon pictus*) and roan antelope (*Hippotragus equinus*) are relatively undisturbed and roam freely^13^. The majority of the Namibian component is under communal tenure and used by conservancies, commercial enterprises and subsistence farmers^15^. Due to resource availability, favourable rainfall and soil conditions for cropping and grazing of livestock, population density in north-central Namibia along the Okavango River and in the Zambezi Region is higher than in the rest of the country. In the northern rural areas, people live in small localised villages in mobile homes or single quarters spread across the landscape^15^. Most households in the region depend directly on natural resources for survival. Agriculture is the most important livelihood strategy and strongly determines the food security and income of the region^16^.

The North-East Parks (NEP) of Namibia make up the geographical core of the KAZA TFCA and provide important migration routes for large herds of African buffalos (*Syncerus caffer*), elephants (*Loxodonta africana*), roan and sable antelopes (*Hippotragus niger*)^13^. However, the area of the North-East Parks and its surrounding conservancies is too small to adequately address the spatial requirements that specific taxa and associated sub-populations need to sustain these populations in the future^13^. A common challenge in the Namibian component and all KAZA signatory country is a high level of human-wildlife conflict.

## Material & Methods

Human–wildlife conflict is an ecological challenge that is strongly influenced by social factors and perceptions, for example community tolerance to wildlife and the value of wildlife to people. The KAZA TFCA is a socio-political factor possibly impacting and changing this interaction.

To address the complexity of different ecological, physio-geographic and socio-cultural, anthropogenic factors affecting wildlife, people and conflict in the region, this study is based on a mixed and multiple method research design incorporating both quantitative and qualitative data collection and analysis methods, using primary and secondary data to support or discard the hypothesis presented above. Most studies on TFCAs in southern Africa focus on social science issues. Key biological data such as the assemblage of coenoses, spatial and habitat patterns of species, as well as reproduction and population parameters are underrepresented. Thus, the qualitative component of this study is based on the reconstruction of historical wildlife distribution parameters to assess the perception of stakeholders based on the dynamics of the megafaunal coenoses of the region. It aimed to objectively analyse changes in wildlife distribution and abundance over time to assess the range of selected species in the past, which can inform management decisions aimed at opening areas for wildlife to revert back to a more natural state with open landscapes. The expert interviews and community surveys serve to verify findings from the reconstruction.

### Secondary data analysis

In order to assess dynamics of wildlife populations in the Namibian component of the KAZA TFCA and their impact on human–wildlife conflict (e.g. Hypothesis 1), we first mapped selected factors (wildlife distribution and numbers, poverty indices, human population density, land-use) over time using QGIS Version 3.12^14^. Five species are considered for wildlife dispersal and wildlife numbers: (1) the African elephant, (2) the lion, (3) the blue wildebeest, (4) the plains zebra (and (5) the African buffalo. The African elephant and lion are commonly named as conflict-causing species^15^. The plains zebra and the blue wildebeest previously migrated through southern Africa on a large scale^17^ and have considerable ecological significance due to their direct impact on grassland forage and indirect impact on ecosystem processes. They are an important indicator for the movement of other species for example lions and other predators^18–20^. The African buffalo is important as a conflict species because it is an important disease vector^21^.

The time spans used for the historical reconstruction were determined by the availability of data. For the reconstruction of wildlife dispersal areas three main points in time were used: Shortridge (1934), Joubert & Mostert (1975) and the most recent range. The most recent range is based on the Atlasing of Namibia – Atlas of Mammals and Carnivores available on the Environmental Information Service Namibia website. The Atlasing project is a data repository for biodiversity data from previous atlases, museum specimen data, game count information, Event Book System data on human-wildlife conflict incidents, research projects and tracker app records. It combines citizen science with these data sources and is constantly updated and verified. The recent range is based on wildlife sightings between 1993 and 2020. Since no information on past wildlife occurrence had been digitalised for Namibia, the hand-drawn maps from Shortridge (1934) and Joubert & Mostert (1975) were geo-referenced and a new QGIS layer created from the indicated occurrence of species in the maps. Of course, hand drawn maps are biased and do not reflect the exact locations of today. However, they can still provide valuable information to support planning. The density of points used to display the wildlife range does not indicate the frequency of sightings or abundance of wildlife. For the Shortridge occurrence layer, the density of points is an exact replica of Shortridge’s hand-drawn sightings. For the Joubert and Mostert layer, the density arises from the pattern the authors used to georeference  their hand-drawn lines. For the most recent data, the size of the points arises from the data collection method used as monad, pentad and QDS grids as well as latitude-longitude records are included in the data repository. All points have a 5 km buffer as authors assume that the species will also occur within 5 km of the sighting.

A variety of different sources was used for the population size of species. The same sources with the same survey techniques were used as far as possible to improve the comparability of estimates over time and across countries. However, certain time frames, countries or species often have been poorly or not at all assessed. Supplementary Material 1 shows the different estimates and the authors from whom the estimates originate. The available information was collected and visualised using Excel (Microsoft Corporation, Redmond, WA, USA). According to Distefano (2005) and Baldwin (2017), human population growth is one of the main reasons for human–wildlife conflict, especially if areas with high population density and high wildlife density overlap^8,22^. Poverty only indirectly influences human–wildlife conflict. However, human–wildlife conflict can considerably influence poverty by further increasing the vulnerability of rural communities. Information on human population density and poverty indices originate from the population and household surveys of the Namibia Statistics Agency, who also provided data on land-uses. Human–wildlife conflict data from the Event Book System (2001 to 2015) was provided by the Ministry of Environment and Tourism. The Event Book System is a component of Namibia’s CBNRM programme with which communities monitor human–wildlife conflict incidences, wildlife numbers, economic returns, rule infringements, etc.^23^. The available data was imported into QGIS 3.12^14^ and visualised for the entire country to assess changes over time. Detailed information on the type of data used, the data source and how they were processed can be found in Supplementary Material 2.

### Analysis of community and expert perceptions

A mixed-method approach was applied to analyse community and expert perceptions on wildlife, human-wildlife conflict, and opportunities and challenges of the KAZA TFCA. Eleven semi-structured interviews with experts from government, civil society, community-based organisations and the university were conducted on a face-to-face basis. In addition, a questionnaire (n = 68) was prepared for community members and distributed by field staff of local NGOs and the Ministry of Environment and Tourism (MET). Approval of the methods and experimental protocols was sought from Humboldt University, the responsible person in the MET in Namibia, an NGO partner and park wardens in the region where the study was conducted and thus correspond to guidelines and regulations. Both community members and experts interviewed gave their informed consent before the interview or commencing the survey.

#### Expert interviews

A semi-structured interview approach was chosen in order to improve the comparability of responses, while at the same time being able to adapt to the flow of the conversation and to the interviewees’ expertise. The results were then compared and contrasted to the results of the community surveys to explain particular phenomena such as wildlife movement, human-wildlife conflict incidents and connections that might not be visible in isolation of the other method.

A list of 12 questions with a short introduction to the topic and the research proposal was sent to the participants in advance to allow for preparation and thus more structured and conclusive information. This allowed interview partners to prepare documents to support their arguments. The questions for both the questionnaire and interviews are grouped into three broad categories: (1) Perception on Wildlife, Human–Wildlife Conflict and Poaching, (2) Knowledge and Opinion on the KAZA TFCA and (3) Possible Opportunities and Challenges, Hopes and Fears once the TFCA is fully implemented. Issues of poaching and tourism were included for the following reasons: The impoverished Zambezi region suffered from poaching by South African military personnel in the 1980s and 1990s. This has led to heavily reduced wildlife populations. After independence, poaching has declined due to strict penalties and CBNRM^24^. However, poaching is still a problem in the region due to the frequent occurrences of human–wildlife conflict^25^. Poaching often occurs in regions where wildlife is perceived to be a threat to livelihood strategies or in remote areas, where less monitoring takes place and the risk of being caught is reduced. Thus, poaching frequently occurs in areas where both human and wildlife populations are very high^25^. Tourism provides the opportunity to increase income from the sustainable use of natural resources^26^. Wildlife-based tourism is primed by the KAZA TFCA to become a profitable and sustainable alternative to agriculture and to reduce the dependence of rural communities on primary production^27^. In addition, benefits from tourism should provide incentives for rural communities to tolerate wildlife^28^.

The Interview Guide and Survey also included questions on poaching and tourism. The questions in the questionnaire and semi-structured interview slightly differ, as they were designed for different stakeholders. To prepare for the interview an Interview Guide was established that covered probes and the hypotheses (Supplementary Material 3).

Before the interview all interview partners were asked if they agreed to having the interview audio-recorded, whether they wanted to remain anonymous and if they had any further questions on the topic to ensure a common understanding. The interviews lasted between 30 minutes to one hour. All interviews were audio-recorded. Records from the audio-recordings and notes taken during the interview were completed after the interview. In addition, a contextual data sheet (Supplementary Material 3) was used to capture the immediate impression after the interview, the setting of the interview, the location as well as the agreement to audio-recordings or wishes to be anonymous.

Eleven interviews were conducted with 7 locally-operating organisation (including two community-based organisations) and 4 institutions operating at a national level. All local organisations were working within the Namibian component of the KAZA TFCA. Experts were selected based on their active involvement and expertise in human-wildlife conflict and transboundary conservation. Data were collected in six weeks of a field visit in the Namibian component of the KAZA TFCA. A heterogeneous set of interview partners with different backgrounds promised new insights and in-depth information. This made it possible to identify key categories and themes relevant to the research problem. Patterns from a heterogenous set of participants are likely to be of interest.

After transcribing all interviews, the data were cleaned and colour-coded with Excel. The different codes were clustered and summarised into categories. The categories were derived from the data and not predetermined based on a theoretical framework. However, they were influenced by the literature review conducted before the field research. Once the categories had been established, the data were summarised in a table comprising the categories, codes and main statements for each category. Based on this summarised table, patterns and relationships between the different categories were determined and prepositions developed, that were then further refined and rearranged. Some results were also quantified through simple counts.

#### Community surveys

The questionnaire aimed to determine the perceptions and opinions of communities, providing another perspective on human–wildlife conflict, poaching and the KAZA TFCA. Using a questionnaire increased the sample size and allowed for clearer and more simple phrasing. The questionnaire could be self-completed or based on a face-to-face completion by the interviewer. The questionnaire was designed based on the questions and topics covered during the expert interviews (Supplementary Material 4) to get the opinion of the people on the ground living with wildlife every day.

To determine the research design requirements a data requirements table (Supplementary Material 5) was created, which included research questions and objectives. Investigative questions were formulated parallel to the expert interview questions. From these the required variables and level of detail in which these should be measured were defined. The variables were then cross-checked with the literature used during the literature review.

The questionnaires were printed in advance and self-administered by the participants. NGO and Ministry of Environment and Tourism staff distributed and collected the questionnaires. The advantage of this was that staff know the communities and often speak their language. In addition, a much larger area and more diverse set of participants could be covered in a short time. However, although they were briefed beforehand, the data collection process could not be monitored. Two hundred questionnaires were printed and packed into sets of 20 surveys including pens.

Sixty-eight questionnaires were completed by a heterogenous group living within and outside of conservancies. Surveys were completed in 9 conservancies in the Namibian component of the KAZA TFCA and by 13 individuals living outside conservancies. Conservancies are structures under Namibia’s Community-Based Natural Resource Management (CBNRM) programme, which receive ownership rights over wildlife and the right to secure income from wildlife resources on their land^26^. As the primary goal was to collect new insights and triangulate these with the other methods, no representative sample was sought. Thus, data collection was based on a convenience sampling approach. The geographical distribution was considered and most regions of the Namibian component of the KAZA TFCA were covered. However, variation within the population was limited and it can be seen as a pilot to a more structured and detailed study in the future.

Once the surveys were collected a list of codes was developed, which included all questions, an ID, variable name, the type of data recorded, and the degree of measurement and the codes. The data were then entered manually into a data matrix in Excel and imported into SPSS Version 23. After defining the codes in SPSS and making sure that everything had been imported the data were analysed mainly for frequencies. Since most of the collected data were categorical or descriptive, the choice of statistical calculations that could be used was limited. Relationships were explored by cross-tabulating data. When relationships became apparent from the results a null hypothesis was formulated and tested using a Peason’s Chi-Square Test to determine if the variables were associated. The null hypothesis was accepted when a 95% probability of relationship not occurring by chance alone existed (p < 0.05). A number of graphs were created to aid interpretation of the results.

Both the community survey with people living in the area and expert interviews explore the perceptions of people, which are inherently biased. Thus, multiple methods and data sources to support or discard assumptions were used to substantiate the results.

## Results

The following chapter presents the insights gathered from the reconstruction of the historical dimension of wildlife trends and species occurrence gathered from the secondary data (literature and document) analysis followed by the expert interviews and community questionnaires.

### Wildlife and human-wildlife conflict trends

Table 1 presents population estimates of the elephant, blue wildebeest, plains zebra, lion and African buffalo for all KAZA TFCA countries. Elephant populations in Namibia and the other KAZA countries have steadily increased since 1934. Plain’s zebra numbers considerably increased in Namibia, but decreased in Botswana, Zambia and Zimbabwe. While the Namibian lion and buffalo populations have remained at a constant level, lion populations in the other KAZA countries decreased. Buffalo numbers increased in Botswana and decreased strongly in Zimbabwe. After sharp declines in wildebeest populations around 1965, they are recovering in both Namibia and Botswana. A more detailed description of wildlife trends can be found in Supplementary Material 6.

**Table 1:**
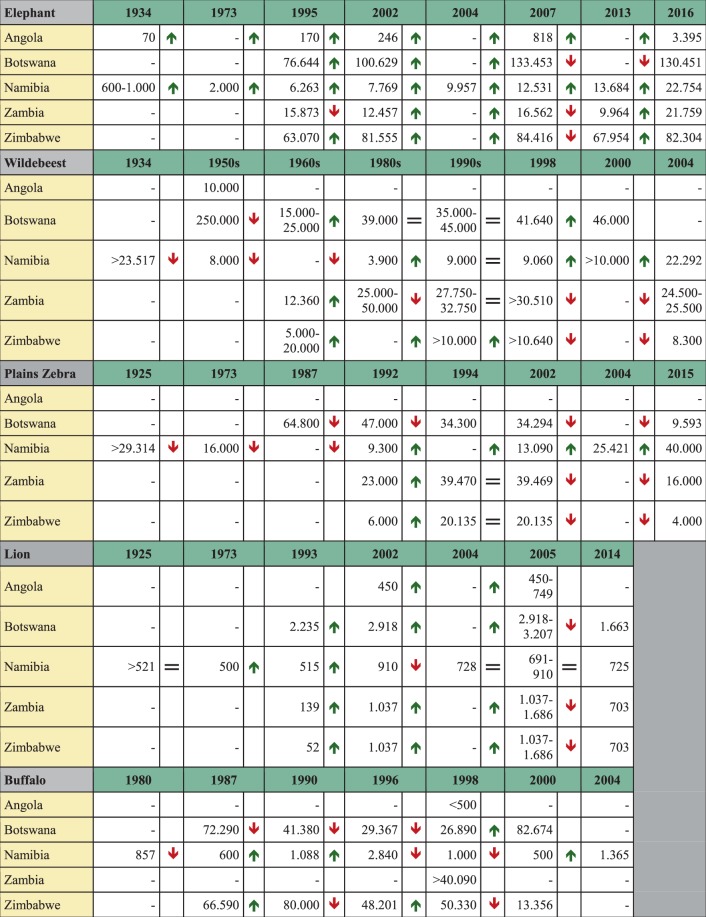
Population estimates of the elephant, blue wildebeest, plains zebra, lion and African buffalo for all KAZA TFCA member states over the years.

Details on the sources of the estimates can be found in Supplementary Material 1.

**Figure 2:**
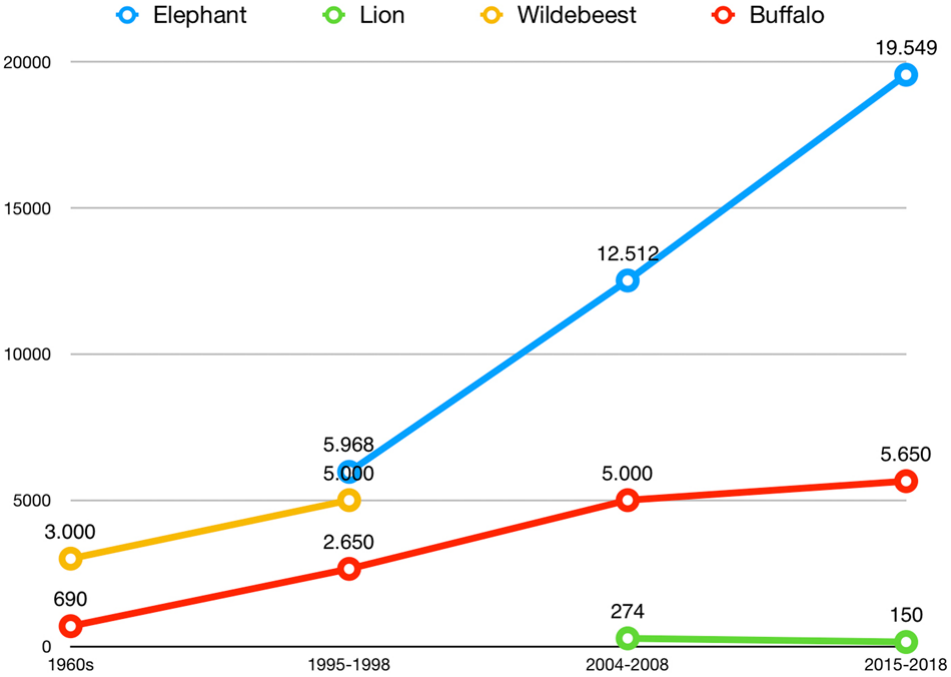
Wildlife trends for the elephant, blue wildebeest, plains zebra, lion and African buffalo in the Namibian component of the KAZA TFCA. The graph depicts the wildlife numbers for four species (elephant in blue, lion in green, wildebeest in yellow and buffalo in red ) in the three regions (Kavango East, Kavango West and Zambezi) that constitute the Namibian component of the KAZA TFCA.

Wildlife numbers in the table above represent species populations for the whole country. Figure 2 shows wildlife trends for four of the five studied species in the Namibian component of the KAZA TFCA. Unfortunately, no disaggregated data by region was available for the Plain’s Zebra and older surveys for the Zambezi and Kavango Regions do not exist. The data from 1995 to 2015/2016 shows that elephant numbers in the Namibian component of KAZA have more than tripled. The region hosts most of Namibia’s elephant population estimated at 22 754 in 2016. Lion populations have declined. However, this estimate for 2018 by the IUCN Cat Specialist Group was not based on new data. Both wildebeest and buffalo populations increased as well. No recent information or aerial surveys have been published since the study of Estes and East in 2009. However, wildebeest seem to recover after they reached their lowest level between the 1960s and 1980s^17^. Buffalo populations have also increased considerably since the 1960s. The estimate of the most recent buffalo population (5650 heads) is based on buffalo sightings during the 2018 conservancy game counts conducted by NACSO in the region. The buffalo population in 2018 is thus likely to be a severe underestimation as it does not account for buffalos living outside of conservancies and conservancies that have not been surveyed.

**Figure 3:**
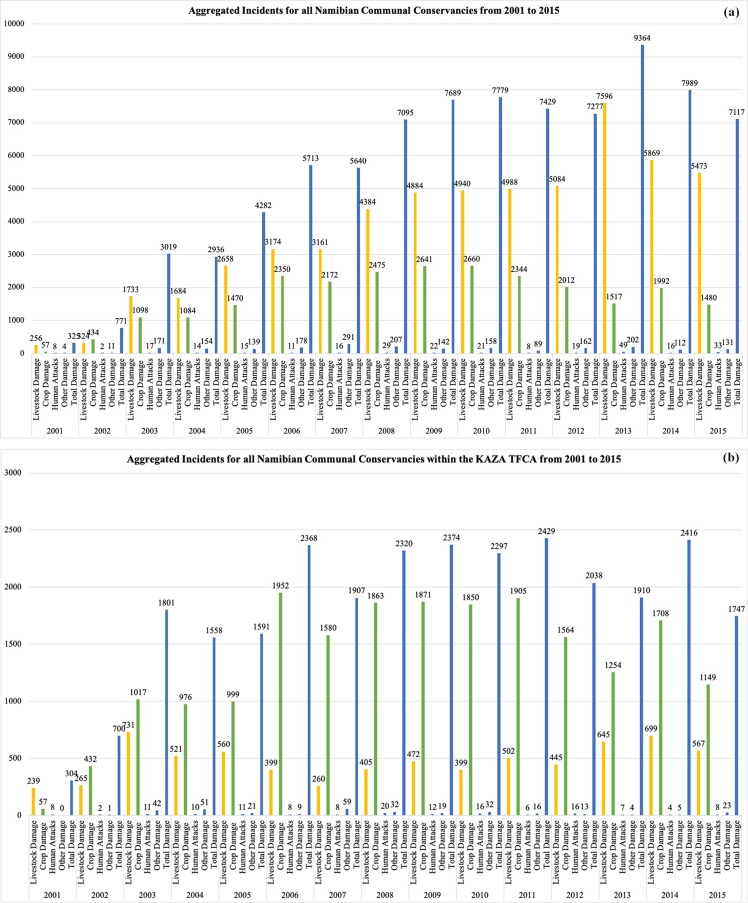
Reported human-wildlife conflict incidents in all conservancies that lie within the KAZA TFCA (n = 21) and in all investigated Namibian conservancies (n = 85). Map (**a**) shows the trend in human-wildlife conflict in all communal conservancies in Namibia between 2001 and 2015, while map (**b**) shows the trend in incidents in the communal conservancies lying within the Namibian component of the KAZA TFCA. The graph shows incidents of livestock damage (yellow), crop damage (green), human attacks (black) and other incidents (dark blue) as well as the total incidents (blue).

Data on reported human–wildlife conflict incidents for all conservancies employing the Event Book System in Namibia and conservancies located in the KAZA TFCA over the last 15 years show different trends. Reported incidences of human–wildlife conflict in all investigated conservancies (Fig. 3) were initially low. From 2003 to 2013 the reported incidents increased and then decreased between 2013 and 2015. Reported incidents in conservancies within the KAZA TFCA (Fig. 3) increased until 2003. Thereafter, incidents remained relatively constant with upward and downward fluctuations. Taking into account all studied conservancies in Namibia, predation of livestock by wildlife is the main challenge. Considering data from the conservancies that lie within the KAZA TFCA alone, crop damage is reported more often (on average around 71% of all reported incidents between 2002 and 2015) than in the Namibian context. Crop damage has decreased in these conservancies since 2012 and livestock predation is increasing. Human attacks and other damages, for example to infrastructure, are much less common (less than 5% of all reported incidents).

### Reconstruction of historical occurrence of selected species

**Figure 4:**
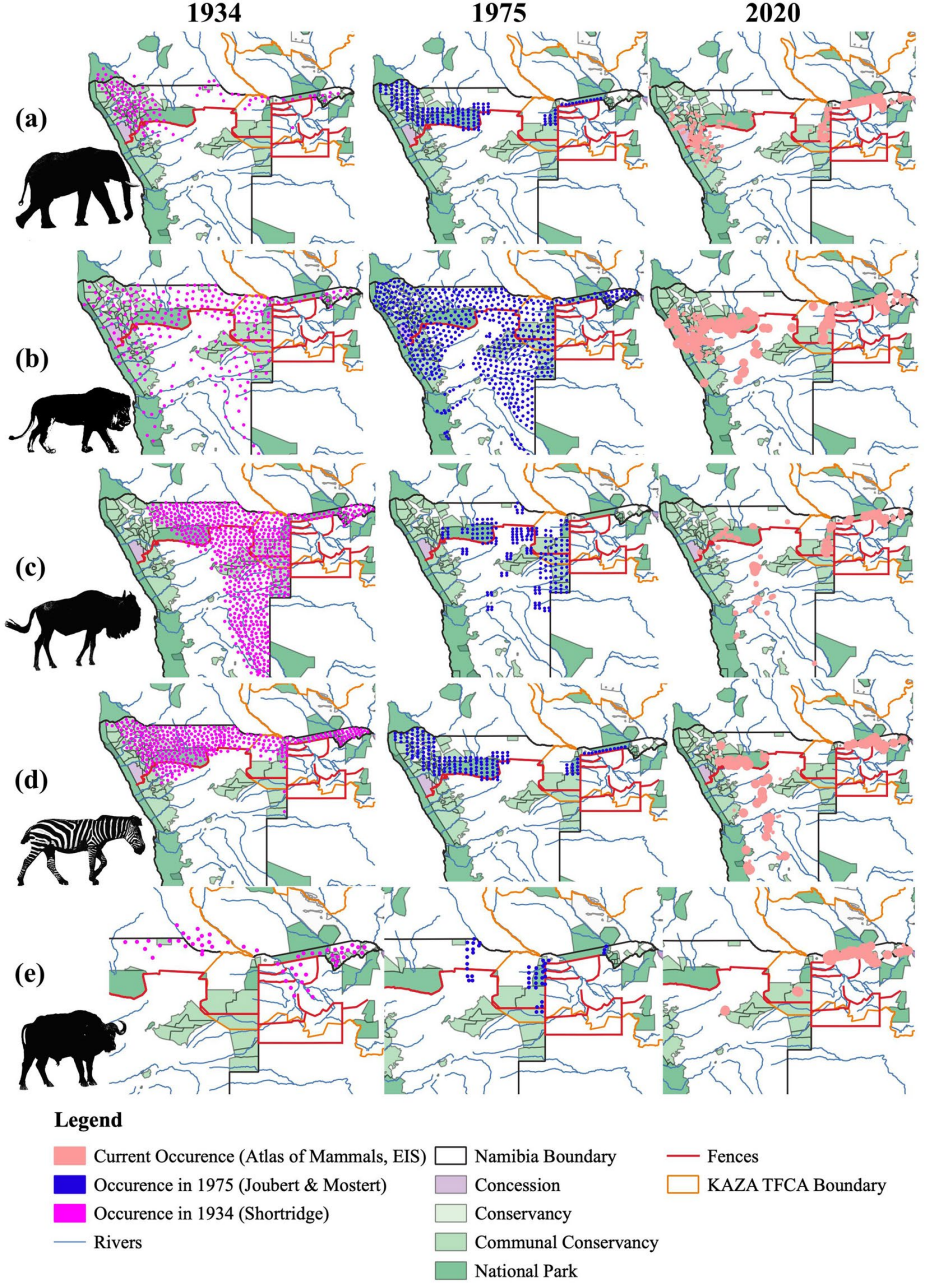
Occurrence patterns of the elephant, lion, blue wildebeest, pains zebra and African buffalo in 1934, 1975 and the most recent range. The map shows the distribution of the elephant (**a**), lion (**b**), blue wildebeest (**c**), plains zebra (**d**) and African buffalo (**e**) in 1934 (purple), 1975 (blue) and the most recent range (pink) indicated in the Atlas of Mammals (Atlasing of Namibia Initiative, Environmental Information Service Namibia) mapped using QGIS 3.12^14^. The size of the points is a result of different grids used for the data collection, which is explained in more detail in the methodology and Supplementary Material 1. In addition, it shows the location of fences (red), rivers (blue) and protected areas (different shades of green with increasing protection).

Figure 4 shows the occurrence patterns of the five selected species in 1934, 1975 and the most recent range from the Atlas of Mammals^29^. Lion and elephant populations expanded their range between 1934 and 1975. Their most recent range is considerably smaller than in 1975 and they are increasingly restricted to protected areas in the Namibian component of the KAZA TFCA and around the Etosha National Park. In the past, the African buffalo had a limited range along the Okavango and the large rivers in the north-east. Today, there are no more buffalos along the Okavango River in north-central Namibia, but they occur all over the north-east. The range of the two migratory species, plain’s zebra and blue wildebeest, was considerably smaller in 1975 than 1934. However, both species were able to recover some of their former range due to reintroductions to farmland and private reserves.

Figure 4 shows larger fences in Namibia and Botswana. Furthermore, the wildlife dispersal areas for 1934, 1975 and the current range based on the Atlas of Mammals and Carnivores are mapped. Fences seem to have a considerable effect on elephant, buffalo and to a certain degree also blue wildebeest. Shortly after the construction of a game-proof fence, the so-called “veterinary cordon fence” or “Red Line in Namibia in 1975, the range of the blue wildebeest was severely restricted, apart from a few smaller populations that survived south of the fence. The Atlas of Mammals also suggests a considerable influence of fences on wildebeest range. The plain’s zebra range also seems to be considerably influenced by fences -especially in the north-east. For both zebra and wildebeest, outliers can be explained by reintroductions on private game farms. The effect of fences on the buffalo population seems to be significant. Buffalo range is cut off along the border fence with Botswana to the west, the Red Line to the south and the Caprivi fence in the Bwabwata National Park. In 1975, elephants occurred south of the Red Line. In the current range elephants are restricted to protected areas (Etosha NP, Khaudom NP, Mangetti NP). Within the Bwabwata National Park their range is cut off along the border with Botswana in the south-western part. Lion range is less influenced by the Red Line in the Etosha/Kunene area, where they occur on both sides of the fence, but is much more restricted in the north-east, where it is cut off by the border fence with Botswana, the Caprivi fence, as well as the Northern and Southern Buffalo fences in Botswana.

### Human component: poverty, population density & land-use

**Figure 5:**
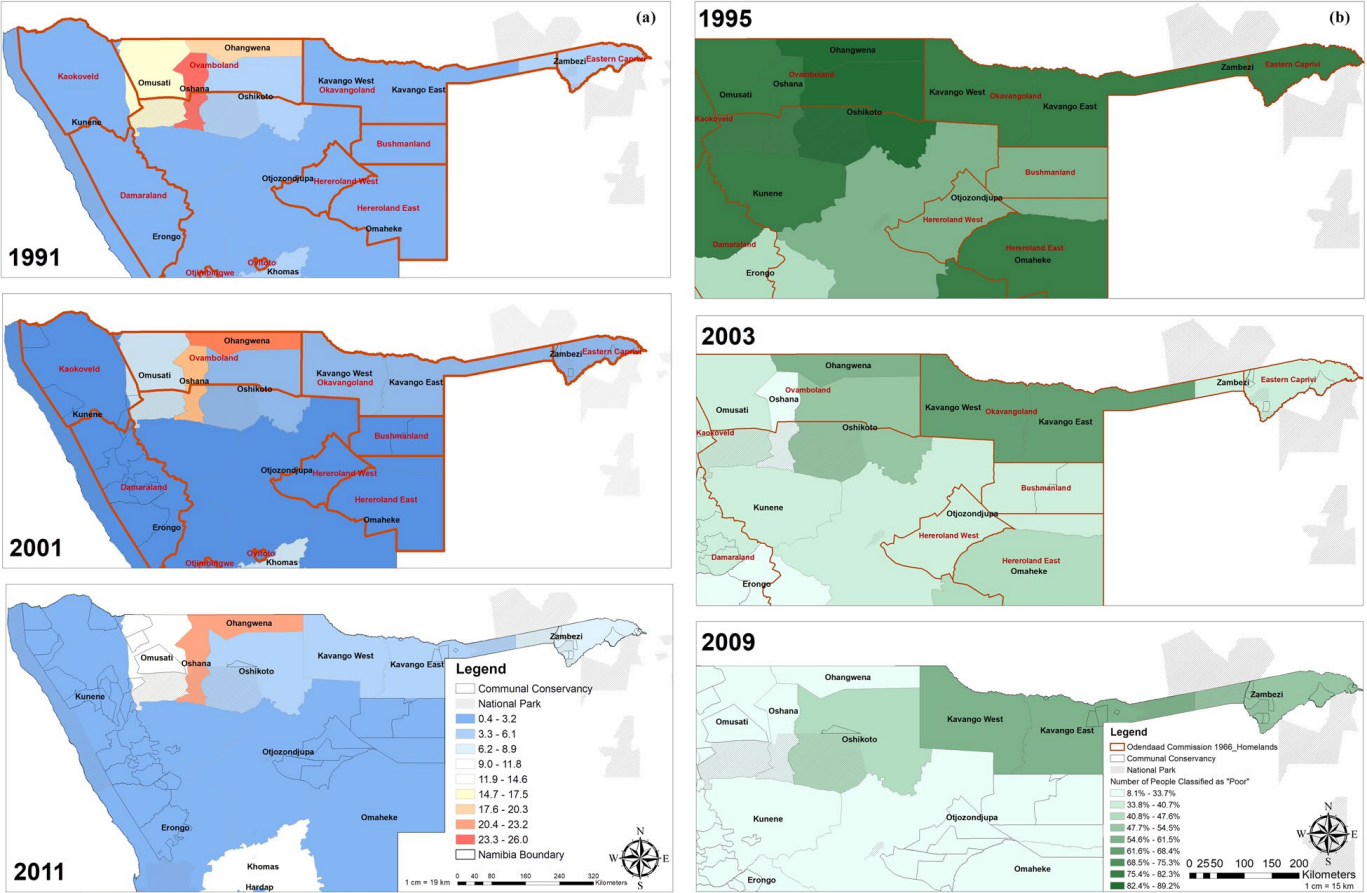
Population density by region in 1991, 2001 and 2011 and the number of people classified as poor by region in 1995, 2003 and 2009. The figures show the population density (**a**) and poverty level (**b**) over time for northern Namibia mapped using QGIS 3.12^14^. Population density is indicated in people per square km: low values are indicated in dark blue and while high values are indicated in dark orange. Black homesteads -to which indigene communities were relocated under South African administration- are outlined in red. The level of poverty (% of people classified as poor) ranges from light green (low) to dark green (high). Light grey areas indicate national parks.

Population density is high in the northern regions of Namibia compared to the rest of the country, especially in some of the former black “homelands” such as Ovamboland, Okavangoland and Eastern Zambezi (Fig. 5). Based on population and housing census data of the Namibia Statistics Agency, there has been an overall increase in population from around 1.4 million in 1991 to around 2.1 million in 2011. As a result, population density has increased across the country. For example, the population density in the Zambezi region has increased from 4.9 inhabitants/km^2^ to 6.2 people/km^2^. Density in the Kavango region increased from 2.7 to 4.6 inhabitants/km^2^ and from 0.9 to 1.4 inhabitants/km^2^ in the Otjozondjupa region^30,31^.

Based on the population censuses of the Namibia Statistics Agency (NSA), the number of people classified as “poor” has been reduced considerably since independence in 1990 (Fig. 5). The proportion of the population classified as poor was reduced in the Kavango (76.3% in 1993/4 to 55.2% in 2011) and Otjozondjupa regions (60.1% in 1993/4 to 33.7% in 2011). The Zambezi region, which had the highest proportion of “poor” people (81.3%) in 1991, also experienced a decrease in 2003/04 (36.5%). However, the proportion increased again to 50.2% in 2011^32^. Although poverty in the Namibian component of the KAZA TFCA has been reduced significantly since independence it remains at a relatively high level compared to the rest of the country.

Displaying data on irrigated areas, livestock density and cropping activity in Namibia shows considerable agricultural activity in the Namibian component of the KAZA TFCA (Fig. 1). High livestock densities (max. 10 livestock units/km^2^ ^33^ occur in the eastern Zambezi region, especially along the border with Zambia. Even within conservancies livestock densities can be high. In the central Zambezi region, a considerable area is under cultivation. Smaller areas with fields can be found all over the region and within conservancies. Two larger irrigated areas can be found in the Zambezi region, one in the Central Zambezi region and one within the Bwabwata National Park. Cattle are allowed within the multiple-use zones of the Bwabwata National Park and communities have small crop fields. Another “hotspot” of agricultural activity is along the border with Angola with relatively high livestock densities and a number of irrigated areas. The rest of the Namibian component has relatively low agricultural activity.

### Expert interviews & community questionnaires

Expert Interviews were conducted with two representatives from the University of Namibia Department for Wildlife Management and Ecotourism, two local and two international NGOs, a government and a KAZA representative, as well as one external consultant and two representatives from community organisations. Two female experts and nine male experts were interviewed. Most participants, who answered the community survey, were between 25 and 35 years (46.3%) and 18 and 25 years (23.9%) old. Thus, the majority of respondents was relatively young and mostly male (64.2%). Ninety-five point one per cent of survey participants were members of a conservancy. The majority of respondents are members of the Kyaramacan Association in Bwabwata National Park (17), Dzoti (7) and Bamunu (7) conservancies. Surveys were also completed in Balyerwa, Kwandu, Mashi, Mayuni, Sobbe and Wuparo. Eleven respondents could not be assigned to a conservancy, either because they were not members or because they did not answer the question.

All experts agreed that the main challenge Namibia is facing is dealing with human–wildlife conflict. This increase was confirmed by communities living in the areas: Sixty per cent of the surveyed community members felt that human–wildlife conflict had increased over the past years, while 32.3% reported a decrease. The most common problems caused by wildlife were crop damage (87.9%) and livestock predation (69.7%). Loss of human life (18.2%) and damage to households (9.1%) were less frequent. The majority only experienced human–wildlife conflict a few times a year (59.1%). Others (18.2%) came into conflict a few times a month or every other month (12.1%). The elephant was stated as one of the main conflict species by 92.3% of respondents, followed by the buffalo (44.6%) and lions (43.1%). While the elephant was seen as a conflict species in every studied conservancy other species were more localised. For example, lions were reported as one of the main species causing major conflicts in Balyerwa, Bamunu, Dzoti, Sobbe and Wuparo, while inhabitants of the Bwabwata National Park, Mashi and Mayuni conservancy did not consider lions as a main conflict causing species in their area.

According to the experts, human–wildlife conflict can be driven by competition for food and space. Livestock is an important investment strategy for many local communities and livestock predation was thus a direct threat to their livelihood and survival. Wildlife, on the other hand, was a collective good. Not every community member benefited and in conjunction with poverty and hunger, this aggravated human–wildlife conflict. Communities, who indicated that they benefited very little, were mainly respondents who did not belong to a conservancy. Most respondents who indicated that they “strongly benefited” from wildlife also indicated that they “strongly valued” wildlife (p = 0.011, N = 61, Value = 21.346, df = 9). Respondents who indicated that they ‘benefit very little’ reported increases in human–wildlife conflict, while 56% of respondents who ‘strongly benefit’ perceived decreasing conflicts (p = 0.011, N = 61, Value = 16.675, df = 6).

All expert interview partners stated that wildlife numbers had increased considerably inside and outside of protected areas over the past years, although considerable numbers were poached. The increasing wildlife numbers reflect the success of the community based natural resource management (CBNRM) programme. This was confirmed by community members: When asked about ecological changes taking place in the region, 68.6% of survey respondents indicated that more wildlife was coming into the area in which they lived. This was especially the case for the Balyerwa, Bamunu, Dzoti, Sobbe and Wuparo conservancies. Groups of a particular species were increasing, according to 57.8% of respondents (17.8% no changes, 24.4% decrease) and species, that had not been in the area before were now coming to the area (56.1%).

According to experts, population density in the Namibian component of the KAZA region is high compared to the rest of the country and more people are moving into the area. Agricultural activities increase with the influx of people. Livestock is often mismanaged spurring further conflict, due to a lack of protective measures especially in an open system without fences. Generally, natural resources are becoming more important for the economy every year and wildlife, through tourism, has become one of the main contributors to the Namibian economy. Wildlife-based activities have a competitive advantage over other land-use activities, since they are better adapted to the (semi-) arid conditions. Community survey results suggest a significant association (p = 0.002, N = 37, Value = 17.146, df = 4) between changes in household income and changes in tourism. Cross-tabulation also confirms that income increased as tourism increased. The importance was likely to increase with the expected impact of climate change.

All interviewed experts were convinced that increasing wildlife and human populations are the main reason for an increase in human-wildlife conflict. However, most (55.1%) of surveyed community members associated increasing human–wildlife conflict with the presence of poachers followed by increasing wildlife populations (46.9%), increasing human populations (22.4%) and water scarcity (18.4%). Thirty of 34 survey respondents (88%) who claimed that poaching increased also reported increases in human–wildlife conflict. Eighteen of 27 (66%) respondents who claimed that poaching decreased reported decreases in human–wildlife conflict (p = 0.000, N = 65, Value = 31.018, df = 4).

According to the interviewed experts, once successfully implemented, the KAZA TFCA could improve the movement of wildlife. Furthermore, connectivity could positively support ecosystem functioning and enhance biodiversity. Communities agreed and believed that the KAZA TFCA would be “very successful” in conserving biodiversity (52.8%), conserving cultural heritage (41.5%), creating a network of interlinking protected areas (52.1%), becoming a prime tourism destination (51.2%) as well as reducing poverty (46.7%). Almost one-third (27.3%) of survey respondents appreciated the concept because it aims to create space for the free movement of wildlife and 20% hoped that KAZA would build capacity in natural resource management, to achieve a more sustainable coexistence of humans and wildlife. Experts suggest that by securing corridors wildlife from areas with very high wildlife densities could disperse into less crowded areas. This would reduce pressure on the land and natural resources and minimise interaction and conflict with people living in areas with high wildlife density. On the other hand, an open and connected system could encourage poaching. In addition, due to the increased movement of wildlife, Namibia could become a wildlife transit route and human–wildlife conflict could increase.

To successfully encourage the dispersal of wildlife across the member countries the conditions within the countries need to be the same. Common ground on veterinary diseases and fences have to be found. In some areas, fences protect wildlife from the invasion of cattle. Social problems would also have to be addressed more seriously to make the concept successful. In addition, ways to control illegal settlement and disease outbreaks have to be found as they spread faster over open borders. Bottom-up planning, awareness campaigns, capacity building and programmes targeting poverty and livelihood enhancement are required to change the attitude of people towards wildlife and the KAZA TFCA. Activities need to be communicated to local communities, so they could see the direct and indirect benefits, e.g. the construction of schools and clinics. This needs to go hand-in-hand with increasing benefits from tourism and hunting and employment opportunities. Local communities should be provided with alternatives to agriculture. Above all, for the KAZA TFCA to become successful, political stability in the region is required and joint strategies to control wildlife and forest crime have to be implemented. Poaching in the region was a transboundary issue and facilitated by open borders, different policies and legislation of the individual countries.

## Discussion

Mapping human-wildlife conflict incidents over time (Fig. 3) supports the assumption of literature that human–wildlife conflict in the Namibian component of the KAZA TFCA is considerable. This is supported by experts and 60% of surveyed community members living in the area.

Reflecting on the results gained, two reasons for a high human-wildlife conflict appear to be key. First, there is a restricted occurrence of species due to anthropogenic influence including land-use decisions, fences and human factors e.g. population growth and poverty. Our study shows that even after wildlife had been severely decimated in the 19th century, wildlife was still more widely dispersed and moved across a larger area than it does today. Records from Van Riebeeck (1653) indicated that elephants existed in considerable numbers as far as the Cape Peninsula and Andersson’s 1858 (cited in Shortridge 1934) records indicated a range as far south as the Tropic of Capricorn^34^. During the 19th century ivory trade boom, elephants had been wiped out in many regions^35^. Commercial hunting and the expansion of settlements have led to declining elephant populations in southern and central Namibia^36^. Lions were systematically poisoned, trapped or shot and considered the least numerous predator in South West Africa by Joubert and Mostert^37^. However, our results show they were still widely dispersed. Today, lions have a very restricted range.

The situation was similar for the other three species presented in the results section. Buffalos were severely decimated during the Rinderpest pandemic of 1896 and subsequent disease control programmes restricting them to zones specifically set aside for wildlife from the 1930s onwards^21,34^. Before that, buffalos had habitually migrated in and out of the present-day Zambezi and Kavango regions forming the Namibian component of the KAZA TFCA. According to Martin^38^, today, the population in the western Zambezi region was isolated from the buffalo population in the east due to the absence of water and could not move into Botswana because of veterinary fences. Settlement and agricultural developments along the Kwando river could possibly isolate the populations in the Bwabwata, Mudumu and Nkasa Rupara National Parks, that could only be linked by movement through Botswana^38^. The erecting of fences contributed significantly to the declining range of many species in the past. Active and abandoned fences restricted wildlife movement^39^ and their range (Fig. 4). Fences were used to limit human–wildlife conflict and in particular the spreading of diseases from wildlife to livestock. Fence construction has shaped many parts of southern Africa and influences the dispersal of wildlife, their habitat and access to food and water.

Based on the results of the reconstruction, all species seemed to be retreating to the north and north-east. Together with increasing wildlife populations, this has led to high wildlife densities in those regions. This is partly caused by historic changes outlined above and anthropogenic influence. According to McKenna (2011), since the Bantu movement in the 1st millennium, which displaced San people in southern Africa, and the European invasion in the 19th century, human population density had been steadily increasing (Fig. 5)^35^. Distefano (2005) and Baldwin (2017) consider an increasing population density as a key driver of human-wildlife conflict especially if wildlife populations are increasing as well (Table 1)^8,22^. The relocation of most of the population into homesteads has led to a considerable reduction in the range of the species present^35^. Although our results do not suggest a direct influence of poverty on human–wildlife conflict or wildlife dispersal areas, it considerably influenced the vulnerability of individuals to human–wildlife conflict and poaching.

Increasing poverty levels in the Zambezi region between 2003/4 and 2011 could be caused by high levels of human–wildlife conflict together with other climate-related factors, which superimpose on existing vulnerabilities: Poverty influences land-use decisions and livelihood strategies. Livelihood strategies dependent on natural resources and subsistence agriculture are threatened by the effects of climate change and poor communities have a limited capacity to adapt to these changes^40^. McKenna believes that people in southern Africa had been living in farming communities in relatively large settlements since 1CE-1000CE, keeping livestock and cultivating crops^35^. Cattle in particular had an important symbolic and material value for these communities^35^. Interview partners confirmed that agricultural activity was intense in northern Namibia and an important contributor to food security in the region. According to Mannetti *et al*.^41^, especially livestock farmers are often opposed to the expansion of protected areas. Together with increasing wildlife and human populations, agricultural expansion and a lack of tolerance for wildlife -due to limited benefits from wildlife- were among the main reasons for human–wildlife conflict^41^.

Secondly, increasing wildlife numbers are restricted to smaller areas increasing wildlife density in the area. Although the range of all species has been declining (Chapter 4.2), wildlife numbers have increased (Chapter 4.1). High wildlife density was one of the main reasons for high human–wildlife conflict mentioned during the interviews. The establishment of protected areas as a response to the collapse of wildlife populations in the past^35^ made them the centre of wildlife movement. Large areas were proclaimed as protected area without considering important seasonal habitats and corridors critical for the migration of species and the functioning of the ecosystem^5^. The restricted range is thus a result of fortress conservation in the past^2^. Interview partners confirmed that after the creation and subsequent shrinkage of protected areas, movement of wildlife across longer distances became rare. Even today, protected areas are a refuge with high wildlife densities as can be seen from the wildlife occurrence maps (Fig. 4). Investigating the situation in Limpopo National Park in Mozambique, Massé (2009) even suggests that the creation of conservation areas based on political decisions can increase human-wildlife conflict, since it shifts power relations and leads to displacement^42^.

According to the interview partners, the success of Namibia’s CBNRM programme, with conservancies acting as shock absorbers to the protected area system, had increased wildlife populations inside and outside of protected areas with wildlife moving freely from unfenced protected areas into the neighbouring conservancies. Communities living within the area confirmed that more wildlife is coming into the area in which they lived and that species that did not occur in the area have established a range. This contributed to the perceived increase in human–wildlife conflict apparent in the community surveys.

Points one and two above confirm Hypothesis 2: historical events shaped the landscape and distribution of wildlife in present day Namibia, which impacted human-wildlife conflict in north-east Namibia. According to Fynn and Bonyongo^5^, some parts of the KAZA TFCA belong to the last functional conservation areas in Africa. Southern Africa has the largest remaining elephant range on the continent. Although this range is only a very small fraction of the area elephants previously inhabited, variations in elephant numbers suggest that cross-border movement between the KAZA signatory countries still existed^36^. In addition, the KAZA countries and South Africa host around a quarter to a third of Africa’s remaining lion population^43^, but some sub-populations were declining rapidly due to increasing population density, the resulting increases in livestock, a lack of prey and the illegal killing of lions^44^. The KAZA TFCA is thus tasked with protecting valuable resources, that are threatened by human-wildlife conflict. The goal is to move away from “fortress conservation” to landscape-level conservation strategies answering calls to enhance ecological connectivity advocated by the recent 13^th^ Conference of Parties to the Convention on the Conservation of Migratory Species of Wild Animals (CMS COP13)^45^.

However, it should be considered that these new strategies must be implemented in areas that have been considerably changed by humans in the past. Wildlife populations became increasingly isolated and restricted to protected areas in all KAZA countries with increasing population density and agricultural expansion around them. Although fences were supposed to limit human–wildlife conflict they can also cause human–wildlife conflict. For example, they might cut elephants off from water sources and force them to break through fences into agricultural land. Badly planned fences could even lead species closer to settlements or agricultural activities^39^. Removing fences, a key requirement for opening landscapes for wildlife and creating ecological connectivity, could have a considerable impact on wildlife distribution, abundance and movement. When the government of Botswana decided to remove sections of the Setata (210 kilometres in 2003) and Nxai Pan Buffalo (66 kilometres in 2004) fences respectively, wildebeest, zebras and elephants quickly re-established previous migration routes^39^. The aim of the KAZA TFCA is to create free movement for wildlife: Interview partners confirmed that this could lead to Namibia becoming a “transit-route” for wildlife thereby increasing human–wildlife conflict (Hypothesis 1). However, it could also open up areas for congested populations to move to. Elephants that mainly occur outside of protected areas, in particular require freedom of movement and could be destructive if concentrated. Larger dispersal areas would allow them to avoid human settlements reducing human–wildlife conflict.

We see three pathways for overcoming key challenges of the KAZA TFCA in opening up landscapes and encouraging the free movement of wildlife. Firstly, to improve the feasibility of wildlife corridors by involving communities: Current strategies focused on the creation of wildlife corridors. If we look at the literature, population numbers and the diversity of species tend to decline if protected areas do not include all functional resource gradients and migratory corridors required to reach seasonal habitats critical for the survival of species^5^. Failure to allow ungulate or other migratory species to migrate seasonally and adapt to temporal and spatial changes in forage quality and quantity, can lead to the cessation of large-scale migrations and has detrimental feedback effects on the ecosystem^5^. According to Naidoo *et al*.^46^, corridors should create connectivity between the protected areas of the involved countries and could increase movement by 50% compared to patches that were not connected^46^. However, interview partners pointed out that corridors needed to be maintained. Since they were not a legal entity community support was essential, and communities had to receive incentives to maintain corridors. Indigenous communities needed to be considered in conservation planning and benefit from wildlife. Community survey respondents who indicated that they ‘benefit very little’ reported increases in human–wildlife conflict. If wildlife became too much of a liability to the already vulnerable livelihoods of people, conserving wildlife would become impossible. Poverty could thus be a threat to biodiversity conservation, the main aim of the KAZA TFCA.

Secondly, to mainstream community-based conservation approaches across all signatory countries: All interview partners and surveyed communities agreed that if all countries created the same environment based on community involvement, KAZA could be a very powerful conservation tool. However, the issue of community involvement was a challenge. The TFCA included five countries and it was difficult to determine who should be involved to which degree. Currently community members felt that biodiversity was more important than people, which had a negative effect on their attitude to wildlife. Wildlife was generally important to many indigenous cultures. However, economic benefits were necessary to change negative attitudes and perceptions of people, who increasingly got into conflict. Both KAZA and the Namibian government tried to control human–wildlife conflict from a political level. One of the main challenges of achieving this on the KAZA level was the differences between the signatory countries. A common approach was being sought for countries with no complementary land uses, different conservation approaches and policies, and who were at entirely different stages of development.

Thirdly, encouraging sustainable development while addressing pressing social needs: Results show that promoting wildlife and regional economic development at the same time is a challenge in itself. The benefits KAZA promised to people only materialise in the long term, which could leave people who expected immediate solutions to social problems disillusioned. Although wildlife-based activities might have a competitive advantage over agriculture, tourism and hunting were currently unable to provide enough benefits even though tourism had recently increased considerably. Hunting could play an important role in generating income for local communities but was largely limited by CITES restrictions and international scrutiny. Elephant export quantities were low given the large population in the region. The degree to which poaching could be controlled would also influence hunting, since elephants had been taken off conservancy’s hunting quotas due to the poaching crisis. Sustainable livestock management would thus have to play a role in conservation efforts due to the importance for local livelihood strategies. Human–wildlife conflict directly affected this livelihood and was thus a social, economic and ecological problem.

## Conclusion

The results show that human–wildlife conflict in the Namibian component of the KAZA TFCA is high and considerably impacts the livelihoods of communities. Although conservation initiatives increased wildlife numbers, the occurrence is more restricted than in times of heavily decimated wildlife populations. Agricultural expansion and the erection of fences to protect the agricultural sector has fragmented wildlife habitat and isolated populations. A trend common to all five countries is that wildlife is increasingly restricted to protected areas that lie within the realm of the KAZA TFCA. The limited occurrence and the increasing restriction to protected areas reduces the resilience of the ecosystem.

One of the key conclusions that can be drawn is the importance to look outside the box, see the bigger picture and consider areas outside of the KAZA TFCA. Areas in -for example- north-central Namibia, the Etosha National Park and other areas in the signatory countries -in which these species used to occur- are important for connectivity, wildlife dispersal and genetic exchange of sub-populations^47^.

To conserve the biodiversity of the region, governments are required to be committed. This also involves the commitment to devolve responsibility and engage the local communities in the planning and management of the area. The government and the local communities must realise that the biodiversity that is left in the region is valuable and needs to be conserved. Without a common understanding on that matter, human–wildlife conflict could be a significant contributor to the failure of the concept.

In addition, our paper shows the importance of integrating social science and biological data in research to better understand large-scale wildlife migrations and human-wildlife conflict. The large area of the KAZA TFCA with its considerable and close to natural megafaunal assemblage is an important case study for the rest of the world regarding the reestablishment of large-scale migrations and the effects of the recovery of predator populations on human-wildlife conflict, that needs to be studied in more detail. This involves looking at other species, considering more socio-economic factors and including climate change impacts on human-wildlife conflict.

Based on this study, further analysis of former occurrence and migration patterns in Namibia and other areas of the world should be conducted. Adopting a cross-disciplinary approach considering ecological, social, anthropogenic and geographical aspects and how these changed over time as it has been applied in this study can provide important insights and innovative solutions to human-wildlife conflict and park management. This demands for more interdisciplinary as well as transdisciplinary approaches to generate a better system-based understanding of large-scale and cross-border protected areas and their connected chances and challenges.

## Supplementary information

Supplementary information.

## Data Availability

The datasets generated during and/or analysed during the current study are available from the corresponding author.
