# Supplementary material for: Transfrontier Conservation Areas and Human-Wildlife Conflict: The Case of the Namibian Component of the Kavango-Zambezi (KAZA) TFCA
Source: Sci Rep. 2020 May 14;10:7964. doi: 10.1038/s41598-020-64537-9 (PMC7224369; doi:10.1038/s41598-020-64537-9)
Supplement: Supplementary file 1 — Supplementary information. [file 41598_2020_64537_MOESM1_ESM.pdf]

# Transfrontier Conservation Areas and Human-Wildlife Conflict: *The Case of the Namibian component of the Kavango-Zambezi (KAZA) TFCA*

**Mirja Stoldt*– Thomas Göttert – Carsten Mann– Ulrich Zeller**

7. SUPPLEMENTARY MATERIAL

*Supplementary Material 1*

Table with all wildlife numbers collected for the reconstruction of historical wildlife trends including sources.

| | Angola | Botswana | Namibia | Zambia | Zimbabwe |
|---|---|---|---|---|---|
| **Elephant** | 1934: 70 (Shortridge 1934)<br>1995: 170 (Barnes et al. 1999)<br>2002: 246 (Blanc et al. 2003)<br>2007: 818 (Blanc et al. 2007)<br>2016: 3.395 (Chase et al. 2016) | 1995: 76.644 (Barnes et al. 1999)<br>2002: 100.629 (Blanc et al. 2003)<br>2007: 133.829 (Blanc et al. 2007)<br>2013: 133.453 (Skinner & Milliken 2016)<br>2016: 130.451 (Chase et al. 2016) | 1934: 600-1.000 (Joubert & Mostert 1975)<br>1973: 2000 (Joubert & Mostert 1975)<br>1995: 6.263 (Barnes et al. 1999)<br>2002: 7.769 (Blanc et al. 2003)<br>2004: 9.957 (Barnes et al. 2004)<br>2007: 12.531 (Blanc et al. 2007)<br>2013: 13.684 (Skinner & Milliken 2016)<br>2016: 22.754 (Skinner & Milliken 2016) | 1995: 15.873 (Barnes et al. 1999)<br>2002: 12.457 (Blanc et al. 2003)<br>2007: 16.562 (Blanc et al. 2007)<br>2013: 9.964 (Skinner & Milliken 2016)<br>2016: 21.759 (Chase et al. 2016) | 1995: 63.070 (Barnes et al. 1999)<br>2002: 81.555 (Blanc et al. 2003)<br>2007: 84.416 (Blanc et al. 2007)<br>2013: 67.954 (Skinner & Milliken 2016)<br>2016: 82.304 (Chase et al. 2016) |
| **Lion** | 2002: 450 (Bauer & v.d. Merwe 2004)<br>2005: 450-749 (Bauer et al. 2005) | 1993: 2.235 (Bauer et al. 2016)<br>2002: 2.918 (Bauer & v.d. Merwe 2004)<br>2005: 2.918-3.207 (Bauer et al. 2005)<br>2014: 1.663 (Bauer et al. 2016) | 1925/26: >521(Magistral Papers)<br>1973: 500 (Joubert & Mostert 1975)<br>1993: 515 (Bauer et al. 2016)<br>2002: 910 (Bauer & v.d. Merwe 2004)<br>2004: 728 (Barnes et al. 2004)<br>2005: 691-910 (Bauer et al. 2005)<br>2014: 725 (Bauer et al. 2016) | 1993: 139 (Bauer et al. 2016)<br>2002: 1500 (Bauer & v.d. Merwe 2004)<br>2005: 1.500-3.199 (Bauer et al. 2005)<br>2014: 100 (Bauer et al. 2016) | 1993: 52 (Bauer et al. 2016)<br>2002: 1.037 (Bauer & v.d. Merwe 2004)<br>2005: 1.037-1.686 (Bauer et al. 2005)<br>2014: 703 (Bauer et al. 2016) |

| | Angola | Botswana | Namibia | Zambia | Zimbabwe |
|---|---|---|---|---|---|
| **Blue Wildebeest** | 1960s: 10.000 (Estes & East 2009) | 1959: 250.000 (Estes & East 2009)<br>1965: 15.000-25.000 (Estes & East 2009)<br>1986/87: 39.000 (Estes & East 2009)<br>1994: 35.000-45.000 (Estes & East 2009)<br>1998: 41.640 (East & IUCN/SSC 1998)<br>2002/3: 46.000 (Estes & East 2009) | 1925/26: >23.517 (Magistral Papers)<br>1950s: 8.000 (Estes & East 2009)<br>1980s: 3.900 (Estes & East 2009)<br>1990s: 9.000 (Estes & East 2009)<br>1998: 9.060 (East & IUCN/SSC 1998)<br>2000: >10.000 (Estes & East 2009)<br>2004: 22.292 (Barnes et al. 2004) | 1960s: 12.360 (Estes & East 2009)<br>1980s: 25.000-50.000 (Estes & East 2009)<br>1990s: 27.750-32.750 (Estes & East 2009)<br>1998: >30.510 (East & IUCN/SSC 1998)<br>2004: 24.500-25.500 (Estes & East 2009) | 1960s: 5.000 - 20.000 (Estes & East 2009)<br>1990s: >10.000 (Estes & East 2009)<br>1998: >10.640 (East & IUCN/SSC 1998)<br>2000-2005: 8.300 (Estes & East 2009) |
| **Plains Zebra** | | 1987: 64.800 (East 1997)<br>1992: 47.000 (Hack et al. 2008)<br>1994: 34.300 (East 1997)<br>2002: 34.294 (Hack et al. 2008)<br>2015: 9.593 (Hack et al. 2008) | 1925/26: >29.314 (Magistral Papers)<br>1973: 16.000 (Joubert & Mostert 1975)<br>1992: 9.300 (Hack et al. 2008)<br>2002: 13.090 (Hack et al. 2008)<br>2004: 25.421 (Barnes et al. 2004)<br>2015: 40.000 (Hack et al. 2008) | 1992: 23.000 (Hack et al. 2008)<br>1994: 39.470 (East 1997)<br>2002: 39.469 (Hack et al. 2008)<br>2015: 16.000 (Hack et al. 2008) | 1992: 6.000 (Hack et al. 2008)<br>1995/6: 20.135 (East 1997)<br>2002: 20.135 (Hack et al. 2008)<br>2015: 4.000 (Hack et al. 2008) |
| **African Buffalo** | 1998: <500 (East & IUCN/SSC 1998) | 1987: 72.290 (Winterbach 1996)<br>1991: 41.380 (Winterbach 1996)<br>1996: 29.367 (Winterbach 1996)<br>1998: 26.890 (East & IUCN/SSC 1998)<br>2001: 82.674 (Martin 2004) | 1980: 857 (Rodwell in Martin 2004)<br>1987: 600 (Winterbach 1996)<br>1990: 1.088 (Martin 2004)<br>1996: 2.840 (Winterbach 1996)<br>1998: 1.000 (East & IUCN/SSC 1998)<br>2000: 500 (Martin 2004)<br>2004: 1.365 (Barnes et al. 2004) | 1998: >40.090 (East & IUCN/SSC 1998) | 1987: 66.590 (Winterbach1996)<br>1991: 80.000 (Winterbach1996)<br>1996: 48.201 (Winterbach1996)<br>1998: 50.330 (East & IUCN/SSC 1998)<br>2001: 13.356 (Martin 2004) |

*REFERENCES WILDLIFE TRENDS*


Barnes, R.F.W., Craig, G.C., Dublin, H.T., Overton, G., Simons, W. & Thouless, C.R. African Elephant Status Report 1998-An Update from the African Elephant Database. *Occasional Paper of the IUCN Species Survival Commission No. 22* (1999).

Barnes, J., Lange, G.M., Nhuleipo, O., Muteyauli, P., Katoma, T., Amupolo, H., Lindeque, P. & Erb, P., Preliminary valuation of the wildlife stocks in Namibia: wildlife asset accounts (2004).

Bauer, H., Chardonnet, P. & Nowell, K. Status and distribution of the lion (*Panthera Leo*) in east and southern Africa. Background paper for East and Southern African Lion Conservation Workshop January 2006 Johannesburg (2005).

Bauer, H., Packer, C., Funston, P.F., Henschel, P. & Nowell, K. *Panthera leo*, The IUCN Red List of Threatened Species 2016: e.T15951A115130419. http://dx.doi.org/10.2305/IUCN.UK.2016-3.RLTS.T15951A107265605.en (2016).

Blanc, J.J., Thouless, C.R., Hart, J.A., Dublin, H.T., Douglas-Hamilton, I., Craig, C.G. & Barnes, R.F.W. African Elephant Status Report 2002-An Update from the African Elephant Database. *Occasional Paper of the IUCN Species Survival Commission No. 29* (2003).

Blanc, J.J., Barnes, R.F.W., Craig, C.G., Dublin, H.T., Thouless, C.R., Douglas Hamilton, I. & Hart, J.A. African Elephant Status Report 2007-An Update from the African Elephant Database. *Occasional Paper of the IUCN Species Survival Commission No. 33* (2007).

Chase, M.J. & Griffin, C.R. Elephants caught in the middle: impacts of war, fences and people on elephant distribution and abundance in the Zambezi Strip, Namibia. *African Journal of Ecology Vol. 47*, 223 – 233 (Blackwell Publishing Ltd., 2009).

East, R. Current status of Burchell's Zebra in Africa, with additional information on Grevy's Zebra and Cape Mountain Zebra. IUCN/SSC Equid Specialist Group (1997).

East, R. & IUCN/SSC Antelope Specialist Group. African Antelope Database 1998. *Occasional Paper of the IUCN Species Survival Commission No.21* (1998).

Estes, R.D. & East, R. Status of the Wildebeest (Connochaetes taurinus) in the wild 1967 – 2005. *Wildlife Conservation Society Working Paper No.37* (2009).

Hack, A.M., East, R. & Rubenstein, D.I. Status and Action Plan for the Plains Zebra. In: Moehlman PD (e.d.) Equids: Zebras, Asses and Horses Status Survey and Conservation Action Plan. IUCN Gland, 43-60 (2002).

Hack, M.A. & Lorenzen, E. *Equus quagga*. Supplementary Material. The IUCN Red List of Threatened Species 2008: e.T41013A10386901. http://dx.doi.org/10.2305/IUCN.UK.2008.RLTS.T41013A10386901.en (2008).

Joubert E. & Mostert P.K.N. Distribution patterns and status of some mammals in South West Africa. Division of Nature Conservation and Tourism South West Africa Administration. Windhoek Scientific Society (1975).

Magistral Papers. Distribution of Game in South West Africa. Letters from 1925 and 1926 achieved in the Swakopmund Scientific Society (1925/26).

Martin, R.B. Transboundary Species Project: the Southern Savannah Buffalo. Species Report for the Southern Savannah Buffalo in Support of the Transboundary Mammal Project of the Ministry of Environment and Tourism Namibia facilitated by the Namibia Nature Foundation and the Living in a Finite Environment (LIFE) Programme (2004).

Skinner, D. & Milliken, T. Include all populations of African Elephant *Loxodonta africana* in Appendix I through the transfer from Appendix II to Appendix I of the populations of Botswana, Namibia, South Africa and Zimbabwe. CoP17 Prop. 16 (2016).

Shortridge, G.C. The Mammals of South West Africa - A Biological Account of the Forms occurring in that Region. Volume 1 and 2. Namibia Scientific Society (William Heinemann Ltd., 1934).

Winterbach, H.E.K. The status and distribution of Cape buffalo *Syncerus caffer caffer* in southern Africa. *African Journal of Wildlife Research 1998* ***28 (3)*** (1996).

*Supplementary Material 2*

List of all layers used for the ArcGIS Maps in the results section and how they were created / processed.

| | | |
|---|---|---|
| Distribution | SHORTRIDGE, G.C. (1934): *The Mammals of South West Africa - A Biological Account of the Forms occurring in that Region*, Volume 1 and 2, William Heinemann Ltd., London, Namibia Scientific Society Windhoek | The hand drawn maps were photographed and geo-referenced with ArcGIS. A new Point Shape file identical to the original hand drawn version was created. |
| Distribution | JOUBERT, E. & MOSTERT, P.K.N. (1975): *Distribution patterns and status of some mammals in South West Africa*, Division of Nature Conservation and Tourism South West Africa Administration | The hand drawn maps were photographed and geo-referenced with ArcGIS. A new Point Shape file identical to the original hand drawn version was created. |
| Distribution | Atlasing of Namibia, *Atlas of Mammals & Atlas of Carnivores*, Environmental Information Service Namibia, http://www.the-eis.com/atlas/. Downloaded February 2020 | The tables were rearranged by species and grid system and exported as CSV. The CSV File was loaded into QGIS and joined with the monad grid or QDS grid based on the grid system used. Species recorded with latitude and longitude were imported separately. The CSV files was "joined" with the grid and symbology changes to display individual fields/points. A 5km (0,05) buffer with segment 16 and mitre 2 was added to better reflect the range. |
| Localities | Namibia Statistics Agency (2001): Localities, Available from https://digitalnamibia.nsa.org.na (Downloaded November 2017) | Using the "select" tool I only mapped the localities that I mention in the relevant part within the Master thesis. |
| Southern Africa Rivers | ROBERTSON, T. (2004): Selection of larger rivers south of the equator, In: Okavango River: The flow of a lifeline. Origin: Digital Atlas of Africa produced by United States Geological Survey. Available from www.the-eis.com (Downloaded November 2017) | This layer was not edited in any way except for the "Symbology". |
| Namibia Boundary | Ministry of Agriculture, Water and Rural Development, Department of Water Affairs, Div. Geohydrology, Hartmut Strub used in the project "Hydrogeological Map of Namibia | This layer was not edited in any way except for the "Symbology". |
| Odendaal Commission 1966 | MENDELSOHN, J. (2002): *5.13 the Odendaal Commission's 1964 Proposals*, In: Chapter 5 Land and History in MENDELSOHN, J., JARVIS, A., ROBERTS, C and ROBERTSON, T. (2002): *Atlas of Namibia: A portrait of the land and its people,* David Philip Publishers, Cape Town, South Africa. Downloaded from the EIS Portal 15 January 2018 | This layer was not edited in any way except for the "Symbology". |
| Regions | Ministry of Land Reform (2013): Regions, Namibia Statistics Agency, Available from https://digitalnamibia.nsa.org.na (Downloaded November 2017) | This layer was not edited in any way except for the "Symbology". |

| | | |
|---|---|---|
| KAZA Protected Areas | IUCN and UNEP-WCMC (): The World Database on Protected Areas (WDPA) online, downloaded in November 2017. Cambridge/UK: UNEP-WCMC, Available at: www.protectedplanet.net/<br>Angola: Protected Areas of Angola, Ministério do Ambiente, 2014; Botswana: Protected Areas of Botswana,"Department of Wildlife and National Parks, 2013; Namibia: Protected Areas of Namibia, Ministry of Environment and Tourism; Zambia: Protected Areas of Zambia,Department of National Parks and Wildlife; Zimbabwe: Protected areas of Zimbabwe,"Forestry Commission, Zimbabwe" 1993 | Shapefiles were downloaded for all individual countries and then intercepted with the KAZA boundary and merged into one layer. |
| KAZA Boundary | Peace Parks (2015): KAZA Transboundary Conservation Area, Available from www.the-eis.com (Downloaded November 2017) | This layer was not edited in any way except for the "Symbology". |
| Event Book System Data | HWC Incidents reported to conservancy Event Book Systems, received from Greenwell Matongo, Ministry of Environment and Tourism | Prepared in Excel. Incidents sorted by type of damage incurred and total damage for every year. Assigned to KAZA protected area layer. Displayed with Symbology Quantities. |
| MET Data | HWC Incidents reported to the Ministry of Environment and Tourism in 2016 and 2017, received from Greenwell Matongo | Not imported into ArcMap. Only analysed with Excel. |
| Livestock Density | Ministry of Agriculture, Water and Forestry (2001): Livestock density per square km, Namibia Statistics Agency, Available from https://digitalnamibia.nsa.org.na (Downloaded November 2017) | This layer was not edited in any way except for the "Symbology". |
| Communal Crop Fields | Ministry of Land Reform (2011): *Communal Crop Fields 2011, Namibia Statistics Agency,* Available from https://digitalnamibia.nsa.org.na (Downloaded November 2017) | This layer was not edited in any way except for the "Symbology". |
| Irrigation Areas | Ministry of Agriculture, Water and Forestry (2001): Livestock density per square km, Namibia Statistics Agency, Available from https://digitalnamibia.nsa.org.na (Downloaded November 2017) | This layer was not edited in any way except for the "Symbology". |
| Large Scale Irrigation Areas | Ministry of Agriculture, Water and Forestry (2001): Livestock density per square km, Namibia Statistics Agency, Available from https://digitalnamibia.nsa.org.na (Downloaded November 2017) | This layer was not edited in any way except for the "Symbology". |
| Population & Household surveys | CENTRAL STATISTICS OFFICE (1994): *1991 Population and Housing Census Basic Analysis with Highlights*, National Planning Commission, Windhoek, Available from https://nsa.org.na/page/data-portal/ (Downloaded November 2017) | Population and housing data was prepared in Excel for all the years available and sorted by region. The information was then merged to the attribute table of the "Regions" layer and density visualised with the "Categories" function. |

| | | |
|---|---|---|
| Population & Household surveys | CENTRAL STATISTICS OFFICE (2003): *2001 Population and Housing Census Basic Analysis with Highlights*, National Planning Commission, Windhoek, Available from https://nsa.org.na/page/data-portal/ (Downloaded November 2017) | Population and housing data was prepared in Excel for all the years available and sorted by region. The information was then merged to the attribute table of the "Regions" layer and density visualised with the "Categories" function. |
| Population & Household surveys | NAMIBIA STATISTICS AGENCY (): *Namibia 2011 Population & Housing Census Main Report*, Windhoek, Available from https://nsa.org.na/page/data-portal/ (Downloaded November 2017) | Population and housing data was prepared in Excel for all the years available and sorted by region. The information was then merged to the attribute table of the "Regions" layer and density visualised with the "Categories" function. |
| Population & Household surveys | NAMIBIA STATISTICS AGENCY (2012): *Poverty dynamics in Namibia: A comparative study using the 1993/94,2003/04 and the 2009/10 NHIES surveys*, Windhoek, Available from https://nsa.org.na/page/data-portal/ (Downloaded November 2017) | Population and housing data was prepared in Excel for all the years available and sorted by region. The information was then merged to the attribute table of the "Regions" layer and density visualised with the "Categories" function. |

*Supplementary Material 3*

# Expert Interview Guide

## Small Introduction

- **Broad Topic:** Analyse the possible Impact the KAZA TFCA could have on the Namibian component of the park.

- **Current strategy:** improving connectivity towards the vision of the KAZA TFCA, actively connecting protected areas establishing corridors for wildlife.

- **Analyse:** effects this increase in connectivity has on wildlife migration and thus human-wildlife conflict. How it changes the dynamics of the region and the impacts on the communities and the economy of the region.

I assume 2 possible Scenarios:

- **Scenario 1:** The implementation of the KAZA TFCA can reduce HWC; wildlife reestablishes old migration routes, that lead them away from settlements. The overall density of populations in a single area is reduced.
- **Scenario 2:** Due to cooperation, removal of barriers and a more stable political climate in member countries, wildlife reestablishes former dispersal areas e.g. in Southern Angola. This leads to more wildlife traveling through Namibia leading to more frequent occurrences of human-wildlife conflict.

- **Context:** 2nd part of my research. 1st part deal with the reconstruction of wildlife migration and abundance under human influence (determine 'natural state' —> predict dynamics)

- Goal of this part: Insight into current situation
  - Challenges
  - Standing / importance of wildlife and the role of HWC
  - Perceptions on KAZA (reasons for the establishment, opportunities and challenges)

## Formality

- Do you agree to the interview being audio-recorded?

*The transcripts of this interview will be transcribed and attached to the thesis. The thesis will be archived in the library of the Humboldt University of Berlin. However, your name can remain anonymised and can be deleted from the transcripts.*

- Do you wish to be anonymised in the thesis?
- Do you have any questions before we start the interview?

| Interview Question | Topics | HT | Probes |
|---|---|---|---|
| **Part 1: Perception on Wildlife, Human-Wildlife Conflict and Poaching** | | | |
| What is the main importance of wildlife for Namibia? | Intrinsic, economic, social | 1 | Why? |
| What benefits do people derive from living with wildlife? | Intrinsic, economic, social | 1 | |
| What challenges are experienced due to the presence of wildlife? | HWC, intrinsic value | 1 | |

| | | | |
|---|---|---|---|
| Do you believe conflict with wildlife has increased or decreased? | HWC | 1 | Why has it increased/decreased? |
| How severe is the poaching threat in the Namibian component of the KAZA TFCA? Do you believe poaching has increased or decreased? | HWC, poaching | 1 | Why has it increased/decreased? |
| **Part 2: Knowledge and Opinion on the KAZA TFCA** | | | |
| What is your opinion on TFCA and the KAZA TFCA? (Panacea idea replacing other concepts) | KAZA | | Do you believe it is a positive or negative development? Why? |
| What do you believe is the main function or the main reason for the implementation? | KAZA | 1, 3, 4, 5 | |
| Do you believe the KAZA TFCA can contribute to:<br>• biodiversity conservation?<br>• conservation of cultural heritage?<br>• creating a network of interlinked protected areas?<br>• becoming a prime tourism destination?<br>• reduce poverty and improve livelihoods? | KAZA , HWC | 1, 3, 4, 5 | • What could limit the achievement of the above objectives?<br>• Do you believe that the achievement of these objectives is realistic? |
| **Possible Opportunities and Challenges** | | | |
| Ecological: What possible ecological consequences might the implementation of the KAZA TFCA have on protected areas, conservancies and community forests in Namibia? | | 3, 4, 5 | • Wildlife movement?<br>• Species abundance and diversity?<br>• Is human-wildlife conflict likely to increase / decrease?<br>• Poaching? |
| Socio-political: How could the communities be affected by the KAZA implementation? | | 1, 2 | • More positive or negative effects?<br>• Could it have positive or negative impacts on tourism?<br>• Contribute to diversifying livelihood strategies? To what degree can it influence household income?<br>• How could it influence community perceptions on wildlife? |
| Economic: What are possible economic consequence on the region? | | 1, 5 | • Could it lead to land-use change in favour of wildlife-based tourism?<br>• Could it contribute to poverty reduction and regional development? |
| How likely is the full implementation, joint management and removal of all fences? | Prospects | | What pre-conditions are important for the success? |

## Contextual Data Sheet

| | |
|---|---|
| Location of the Interview | |
| Date and Time | |
| Setting of the Interview (quite, noisy, interrupted etc.) | |
| Background Information on the Participant | |
| Immediate Impression | |
| Audio-recording | |
| Anonymisation | |

# Transfrontier Conservation Areas and Human-Wildlife Conflict: Cause or Cure?

## The Case of the Kavango-Zambezi (KAZA) Transfrontier Conservation Area and its Effects on Namibia's North-East Parks

Mirja Stoldt
Master of Science Student
Humboldt University Berlin, Germany
stoldtmi@student.hu-berlin.de; 081-2883401

Dear valued community member,

This questionnaire is part of my master thesis for a Master of Science degree in "Integrated Natural Resource Management" at the Humboldt University of Berlin. My master thesis deals with possible effects the establishment of the Kavango-Zambezi (KAZA) Transfrontier Conservation Area might have on human-wildlife conflict in the communal lands and conservancies surrounding the North-East Parks of Namibia. To get a better understanding of the problem and community perceptions, your responses are highly valued and important to me.

Your decision to take part in this questionnaire is voluntary. If you decide to take part, it should take you around 15min to complete all three parts of the survey. If you require any support please feel free to approach me. We can also go through the questionnaire together. Please answer the questions in the space provided and feel free to add any comments you might have.

The information you provide will be treated in the strictest confidence, thus you are not asked to provide your name or address anywhere. I hope you will enjoy completing the questionnaire.

Thank you very much for contributing to the successful completion of my Masters degree, your input, knowledge and time is highly valued.

Many thanks for your help,

Mirja Stoldt

## Demographic Information

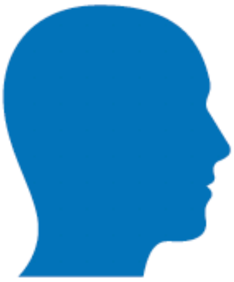

1. Age:
2. Gender:
3. Marital status:
4. Occupation:
5. Region/Constituency/Village: ____________________
5. Conservancy Member: yes / no
6. Conservancy Name: ____________________

## Part 1: Perception on Wildlife, Human-Wildlife Conflict and Poaching

**1.1. How much do you value wildlife?**

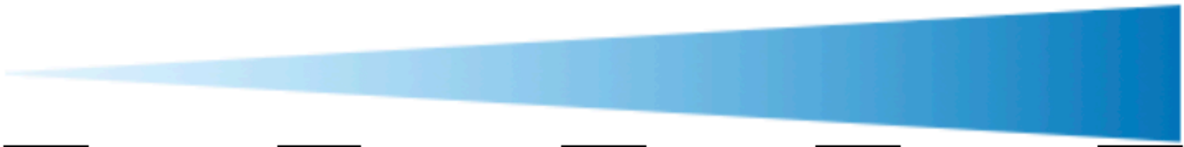

| 1 | 2 | 3 | 4 | 5 |
|---|---|---|---|---|
| I do not value wildlife. | I kind of don't value wildlife. | I am indifferent. | I kind of value wildlife. | I strongly value wildlife. |

**1.2. Why or why don't you value wildlife? *Please note down the first points that come to your mind.***

____________________

____________________

____________________

____________________

**2.1. Do you benefit from wildlife?**

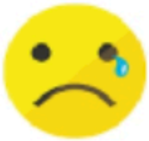
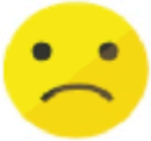
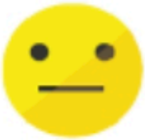
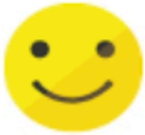
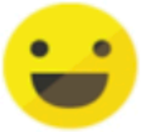

| 1 | 2 | 3 | 4 | 5 |
|---|---|---|---|---|
| I do not benefit at all. | I benefit very little. | I don't know. | I kind of benefit. | I strongly benefit. |

**2.2. What benefits to you get from wildlife?** ***Please tick ✓ all answers that apply to you.***

| | |
|---|---|
| 1 | Meat |
| 2 | Income from Tourism |
| 3 | Income from the Conservancy |
| 4 | Cultural heritage / importance |
| 5 | Other (please specify) ____________________ |

**3.1. Do you want to live with wildlife?**

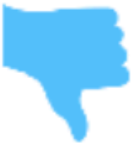

1

I want wildlife to disappear.

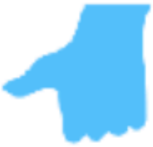

2

I don't really mind living with wildlife.

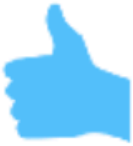

3

I want to live with wildlife.

**3.2. I consider wildlife to be…**

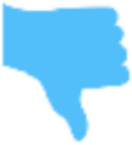

1

…a threat.

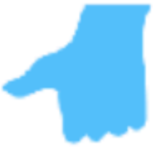

2

I don't know.

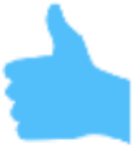

3

…an asset.

**3.3. What are the main problems you experience with wildlife?** ***Please tick ✓ all answers that apply to you.***

| | |
|---|---|
| 1 | Crop damage |
| 2 | Killed livestock |
| 3 | Fear for my and my family's life |
| 4 | Damage to my home. |
| 5 | Other (please specify) ______________________ |

______________________

**4.1. Do you believe <u>conflict with wildlife</u> has increased, remained the same or decreased?**

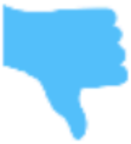

1 Decreased.

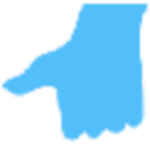

2 About the Same.

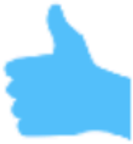

3 Increased.

**4.2. How often are you affected by human-wildlife conflict?**

| 1 | 2 | 3 | 4 | 5 | 6 | 7 |
|---|---|---|---|---|---|---|
| Every day. | Every other day. | Every week. | A few times a month. | Every other month. | A few times a year. | I haven't come into conflict. |

**Human-wildlife conflict** is *"when wild animals injure, destroy or damage human life or property and are killed, injured, captured or otherwise harmed as a result -i.e. both humans and animals suffer from the interaction with each other."* (WWF 2008: p. 15)

**4.3. Which species do you mostly get into conflict with?**

______________________________________________

______________________________________________

______________________________________________

**4.4. Which species do you fear the most?**

______________________________________________

______________________________________________

______________________________________________

**4.5. What are possible reasons for an increase in human-wildlife conflict?** ***Please tick all answers that apply to you.***

***Please only answer this question if you believe human-wildlife conflict has increased!***

- [1] More people.
- [2] More wildlife.
- [3] New wildlife species.
- [4] Poachers.
- [5] Water scarcity.
- [6] Barriers *(please specify)* ______________________
- [7] Other *(please specify)* ______________________

**5.1. Do you believe <u>poaching</u> has increased, remained the same or decreased?**

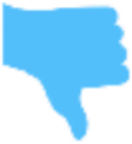

[1] Decreased.

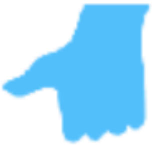

[2] About the Same.

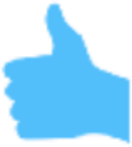

[3] Increased.

**5.2. Have you ever seen or heard of <u>poaching</u> in your environment?**

| 1 | 2 | 3 | 4 | 5 | 6 | 7 |
|---|---|---|---|---|---|---|
| Never. | A few times a year. | Every other month. | A few times a month. | Every week. | Every other day. | Every day. |

**5.3. What are possible reasons for an increase in <u>poaching</u>? *Please tick ✓ all answers that apply to you.***

***Please only answer this question if you believe poaching has increased!***

| | |
|---|---|
| 1 | More people. |
| 2 | More wildlife. |
| 3 | New wildlife species. |
| 4 | Lack of income / poverty. |
| 5 | Do not care about wildlife. |
| 6 | Other *(please specify)* ______________________ |

**Do you have any remarks or thoughts you want to share concerning wildlife, human-wildlife conflict or poaching. Please note them here! If not please move on to Part 2.**

## Part 2: Knowledge and Opinion on the Kavango-Zambezi (KAZA) Transfrontier Conservation Area

**6.1. Have you heard of the Kavango-Zambezi (KAZA) Transfrontier Conservation Area?**

| | | | |
|---|---|---|---|
| 1 | Yes. | 2 | No. |

A **transfrontier conservation area (TFCA)** is *"a relatively large area that straddles the boundaries of two or more countries, and covers large-scale natural systems, encompassing one or more protected areas as well as multiple resource areas"* (DRAPER ET AL. 2004)

- It is the largest transboundary conservation area in Southern Africa and the world and encompasses parts of Namibia, Angola, Botswana, Zambia and Zimbabwe. (KAZA TFCA SECRETARIAT 2010)
- The TFCA should contribute to peace and stability within the region, establish links between fragmented habitats and create economic development through tourism once it is fully implemented (MUNTHALI 2007).

**6.2. How have you heard of of the Kavango-Zambezi (KAZA) Transfrontier Conservation Area?**

***Please tick all answers that apply to you.***

| | |
|---|---|
| 1 | Radio |
| 2 | TV |
| 3 | Newspaper |
| 4 | Conservancy |
| 5 | Other community members |
| 6 | Government officials |
| 7 | Non-Governmental Organisations (NGOs) |
| 8 | Other *(please specify)* ______________________ |

**6.3.1. Do you like the idea?**

| | | |
|---|---|---|
| 1 | 2 | 3 |
| No. | I don't know. | Yes. |

**6.3.2. Why do you like the idea? Why don't you like the idea?**

______________________________

______________________________

______________________________

______________________________

**6.4. What do you think were the main reasons for creating the KAZA TFCA?**

***Please tick ✓ all answers that apply to you.***

| | |
|---|---|
| 1 | Peace, political cooperation, political stability. |
| 2 | Biodiversity conservation and create more space for wildlife. |
| 3 | Harmonising conservation practices across countries. |
| 4 | Poverty reduction and regional development. |
| 5 | Conserving cultural heritage and reestablishing cultural ties. |
| 6 | Expansion of tourism. |
| 7 | Diversifying livelihood strategies. |
| 8 | Other *(please specify)* ______________________________ |

**6.5. Do you believe the KAZA TFCA can be successful at:**

| | Not successful. | Maybe successful. | Not sure. | Most probably successful. | Very successful. |
|---|---|---|---|---|---|
| 6.5.1. Biodiversity conservation. | 1 | 2 | 3 | 4 | 5 |
| 6.5.2. Conservation of cultural heritage / reuniting cultures. | 1 | 2 | 3 | 4 | 5 |
| 6.5.3. Creating a network of interlinked protected areas. | 1 | 2 | 3 | 4 | 5 |
| 6.5.4. Become a prime tourism destination. | 1 | 2 | 3 | 4 | 5 |
| 6.5.5 Reduce poverty and improve livelihoods. | 1 | 2 | 3 | 4 | 5 |

## Part 3: Possible Opportunities and Challenges, Hopes and Fears

**6.6. Have you observed any changes in the landscape / nature / environment around you?**

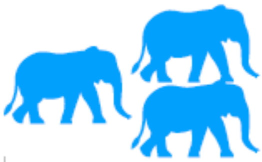

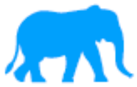

| | | | |
|---|---|---|---|
| 6.6.1. Wildlife movement. | More wildlife is coming here. 1 | No changes. 2 | Less wildlife is coming here. 3 |
| 6.6.2. Species abundance. | Groups of one species are getting bigger. 1 | No changes. 2 | Groups of one species are smaller. 3 |
| 6.6.3. Species diversity. | New types of animals are coming. 1 | No changes. 2 | Some animals are not coming here anymore. 3 |
| 6.6.4. Fences. | New fences have been built. 1 | No changes. 2 | Fences have been removed. 3 |
| 6.6.5. Other *(please specify)* | ______ | | |

**6.7. Have you observed any changes within your community?**

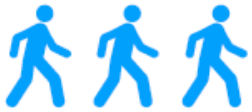

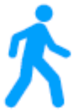

| | | | |
|---|---|---|---|
| 6.7.1. Tourism. | Tourism is increasing. 1 | No changes. 2 | Tourism is decreasing. 3 |
| 6.7.2. Livelihood strategies. | More livelihood strategies based on wildlife. 1 | No changes. 2 | Less livelihood strategies based on wildlife. 3 |
| 6.7.3.Attitude to wildlife. | My attitude has improved. 1 | No changes. 2 | My attitude has gotten worse. 3 |
| 6.7.4. Killed wildlife. | More wildlife is killed. 1 | No changes. 2 | Less wildlife is killed. 3 |
| 6.7.6. Other *(please specify)* | ______ | | |

**6.8. Have you noticed changes in your or others' economic situation?**

| | | | |
|---|---|---|---|
| 6.8.1. Land-use. | More people in agriculture / fishing. 1 | No changes. 2 | More people in wildlife-based activities. 3 |
| 6.8.2. Livelihood Strategy Diversification. | More people with a single livelihood strategy. 1 | No changes. 2 | More people with multiple land-use activities. 3 |
| 6.8.3.Poverty. | More livelihood strategies based on wildlife. 1 | No changes. 2 | Less livelihood strategies based on wildlife. 3 |
| 6.8.4. Household income. | Income has increased. 1 | No changes. 2 | Income has decreased. 3 |
| 6.8.5. Other *(please specify)* | | | |

**6.9.1. Do you believe the implementation is realistic?**

1 **Yes.**

2 **No.**

**6.9.1. Why?** ***Please note any comments you might have.***

| **Research Question:** | If we assume, that the underlying idea of the KAZA TFCA is to create a large area in which wildlife can roam freely, what effects will the implementation of the mega-conservation area have on human wildlife conflict at the heart of the park (Namibia's North East Parks)? | | | |
|---|---|---|---|---|
| **Objective:** | Use these findings to predict possible changes the implementation of the KAZA TFCA may have on the Namibian component, taking into account cultural, economic and ecological factors. | | | |
| **Type of Research:** | Descriptive | | | |
| **Investigative Questions** | **Variable(s) required** | **Detail** | **Theory & Literature** | **Survey** |
| **Part 1: Perception on Wildlife, Human-Wildlife Conflict and Poaching** | | | | |
| What is your opinion/ relationship to wildlife? | General opinion, perceived increase/ decrease, intrinsic & social value | 5 point scale: I value wildlife - I don't value wildlife<br>Open-ended question: Why? | JONES & BARNES 2006: p. 21<br>MOORE 2009: p. 24 | ✓ |
| What benefits do you derive from living with wildlife? | Economic value | 5 point scale: I strongly benefit - I do not benefit at all<br>List: what benefits? | JONES & BARNES 2006: p. 21<br>MOORE 2009: p. 24 | ✓ |
| What challenges do you experience due to the presence of wildlife? | Disadvantages, human-wildlife conflict, loss of income (crops/ LS) | 3 point scale: Want to live with wildlife, indifferent, want wildlife to disappear, more of a threat, more of an asset<br>List: main problems | MULONGA ET AL. 2003: p. 18<br>JONES & BARNES 2006: p. 21<br>WWF 2008: p. 15 | ✓ |
| Do you believe HWC has increased or decreased? Why? | HWC occurence, causes | 3 point scale: Increased, the same, decreased<br>7 point scale: affected by HWC every day - I haven't come into conflict.<br>Open-ended: which species (HWC)<br>Open-ended: most feared species.<br>List: possible reasons. | JONES & BARNES 2006: p. 16 | ✓ |
| Do you believe poaching has increased or decreased? Why? | Poaching occurence, causes | 3 point scale: Increased, the same, decreased<br>7 point scale: Heard of poaching every day. - never heard of poaching.<br>List: possible reasons. | EUROPEAN COMMISSION 2016: p. 42 | ✓ |
| **Part 2: Knowledge and Opinion on the KAZA TFCA** | | | | |
| Have you heard of the KAZA TFCA and what do you associate with it? | Knowledge, perception, opinion, Source | Yes or No.<br>List: how?<br>3 point: like the idea?<br>Open-ended: why? | PEACE PARKS FOUNDATION 2014: p. 26 | ✓ |
| What do you believe is the main reason for the implementation of the KAZA TFCA? | Opinion | List | MURPHY n.d.: p. 7 - 8) | ✓ |
| Do you believe the KAZA TFCA can achieve its objectives? | Conservation, biodiversity, cultural heritage, tourism, poverty, livelihoods | Matrox with 5 pint scale: not successful - very successful. | | ✓ |

| | | | | |
|---|---|---|---|---|
| **Research Question:** | If we assume, that the underlying idea of the KAZA TFCA is to create a large area in which wildlife can roam freely, what effects will the implementation of the mega-conservation area have on human wildlife conflict at the heart of the park (Namibia's North East Parks)? | | | |
| **Objective:** | Use these findings to predict possible changes the implementation of the KAZA TFCA may have on the Namibian component, taking into account cultural, economic and ecological factors. | | | |
| **Type of Research:** | Descriptive | | | |
| **Investigative Questions** | **Variable(s) required** | **Detail** | **Theory & Literature** | **Survey** |
| **Part 3: Possible Opportunities and Challenges, Hopes and Fears** | | | | |
| Have you observed any ecological changes? | Wildlife movement, species abundance & diversity, HWC, poaching, fences | Matrix with 3 point scale: increase, no changes, decrease. | CUMMING 2008: p. 66<br>KARIDOZO ET AL. 2016: p. 16 - 17 | ✓ |
| Have you observed any changes in the community? | tourism, livelihood strategy, household income, community perception | Matrix with 3 point scale: increase, no changes, decrease. | MURPHY n.d.: p. 2 | ✓ |
| Have you observed changes in your and others economic situation? | Land-use change, poverty reduction, regional development | Matrix with 3 point scale: increase, no changes, decrease. | MURPHY n.d.: p. 2 | ✓ |
| Do you believe the implementation is realistic? | Opinion | Yes/no<br>Open-ended: why? | MURPHY n.d.: p. 7 - 8) | ✓ |

*Supplementary Material 6*

**WILDLIFE TRENDS**

*African Elephant (Loxodonta africana)*

Table 1 outlines changes in wildlife numbers for selected species over time. An upward trend in elephant numbers since 1934 can be observed in all KAZA countries. While growth has slowed in Botswana and Zimbabwe, Namibia's population is steadily increasing. According to Barnes et al. [18], around 6263 elephants were resident in Namibia by 1995. The majority occurred in the present-day Zambezi region (4883), the Etosha National Park (1189) and the Khaudom/Tsumkwe region (1085). The same study estimates a definite population of 76 644 for Botswana, 15 872 for Zambia and 63 070 for Zimbabwe. No counts were conducted in Angola. Speculative estimates for the country are around 170 elephants [19]. The elephant population in Namibia continued to increase. The estimate for the whole country was 12 531 in 2007. The population in the present-day Zambezi region almost doubled (8725) and the population in the Khaudom/Kavango region (3787) now exceeded the population in the ENP (2057). Namibia's elephant population further increased to 22 754 elephants estimated for 2015/16 [20]. Increases have been noted for almost all populations: Etosha National Park (around 2911 [21]), Khaudom National Parks and adjacent conservancies (around 6413 [21]) and the Zambezi region (13 136 [22]). Smaller numbers still exist in the Kunene region (314 by [13]) and Mangetti Game Reserve (67 in 2014) [20]. Populations in Angola also increased (3395 in 2016). Numbers slightly declined in Botswana (130 451). After small dips in 2013, populations in Zambia (21 759) and Zimbabwe (82 304) respectively seemed to be increasing and stable [23].

**Elephant numbers in the KAZA TFCA between 1934 and 2016**

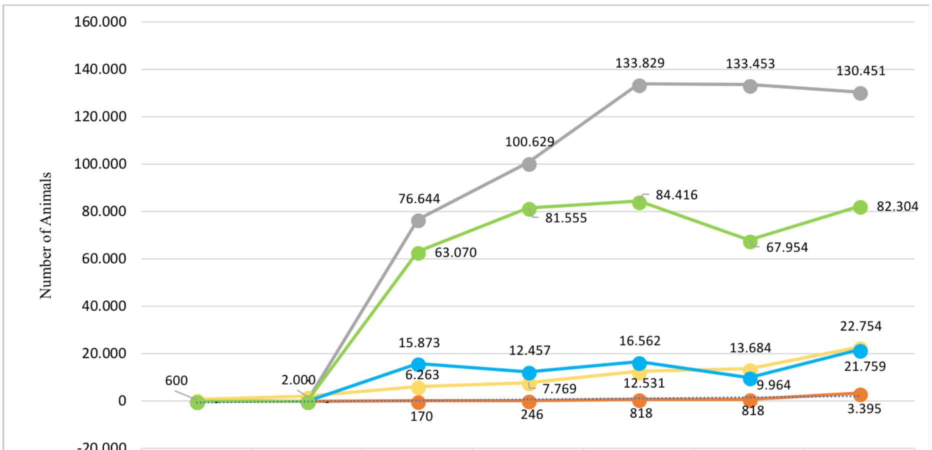


| | 1934 | 1973 | 1995 | 2002 | 2006 | 2013 | 2016 |
|---|---|---|---|---|---|---|---|
| Angola | 70 | - | 170 | 246 | 818 | 818 | 3.395 |
| Botswana | - | - | 76.644 | 100.629 | 133.829 | 133.453 | 130.451 |
| Namibia | 600 | 2.000 | 6.263 | 7.769 | 12.531 | 13.684 | 22.754 |
| Zambia | - | - | 15.873 | 12.457 | 16.562 | 9.964 | 21.759 |
| Zimbabwe | - | - | 63.070 | 81.555 | 84.416 | 67.954 | 82.304 |

*Lion (Panthera leo)*

There were an estimated 521 lions in Namibia in 1925/26 [24]. In 1973, there were an estimated 500 lions in the whole country. Populations seemed to be relatively stable, as Bauer et al. [25] suggested around 515 lions in 1993. At the same time, they estimated around 2235 for Botswana, 139 for Zambia and 52 for Zimbabwe. No information was available for Angola [25]. Namibia's lion population increased strongly between 1993 and 2002 (to 910). Populations in

Botswana (2918), Zambia (1500) and Zimbabwe (1037) also increased. Lion populations in Namibia seemed to stabilise subsequently with estimated populations of 728 in 2004 [19], 691 in 2005 [26] and 725 in 2014 [25]. Lion populations in the other KAZA countries except for Angola (749 in 2005), declined with 1663 lions in Botswana, 100 in Zambia and 703 in Zimbabwe in 2014 [25].

**Lion numbers in the KAZA TFCA between 1926 and 2014**

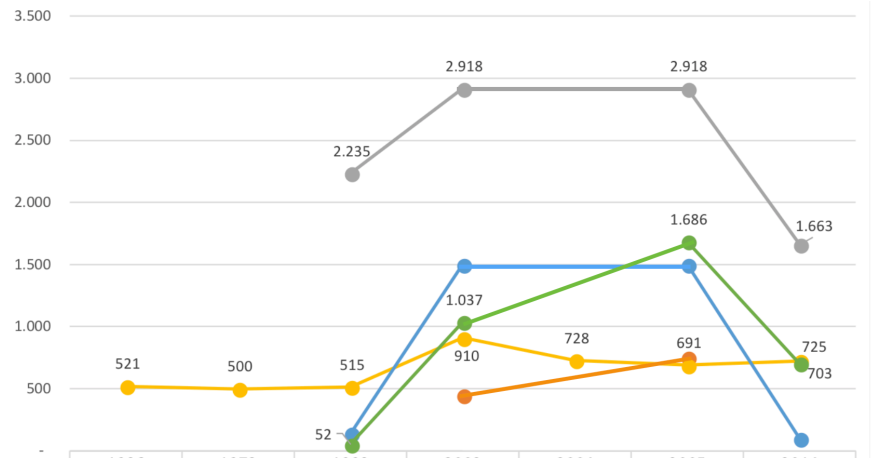


| | 1926 | 1973 | 1993 | 2002 | 2004 | 2005 | 2014 |
|---|---|---|---|---|---|---|---|
| Angola | | | | 450 | | 749 | |
| Botswana | | | 2.235 | 2.918 | | 2.918 | 1.663 |
| Namibia | 521 | 500 | 515 | 910 | 728 | 691 | 725 |
| Zambia | | | 139 | 1.500 | | 1.500 | 100 |
| Zimbabwe | | | 52 | 1.037 | | 1.686 | 703 |

*Blue Wildebeest (Connochaetes taurinus)*

While 23 517 blue wildebeest occurred in Namibia in 1925 [24] they declined to an estimated 8000 in 1967. The population continued to decline to 3900 in the 1980s, of which 2200 lived in the Etosha National Park [27]. Around 9060 occurred in Namibia in the 1990s, of which 3000 were in the Etosha National Park. Populations had increased considerably to 5000, in particular on private farmlands in the north-east. While the ENP populations remained stable (2000 to 3000 in 2002), populations in the rest of the country were increasing and likely exceeded 10 000 wildebeest in 2000 [26]. Barnes estimated 22 292 wildebeest for Namibia in 2004 [19]. The trend has been similar in the other KAZA countries. In the 1960s, Zimbabwe probably had more than 11 000 wildebeest, mostly on private land. Although the numbers were subsequently reduced, they increased again towards the end of the 1990s, exceeding 10 000 [27]. Around 10 650 wildebeest occurred in Zimbabwe in 1995 [28].

In the 1970s, a large Kalahari population of wildebeest had not yet been entirely cut off and approximately 260 000 wildebeest still occurred in the Botswana section. The population declined to 39 000 in 1986/87 [27]. By 1995, the population was stable at 41 650 head [28]. Around 46 000 occurred in Botswana in 2002/03 [27]. Data for Angola is very poor. However, wildebeest seem to have occurred widely. Around 10 000 survived in the southern parts in the 1960s. In 1995, 30 510 wildebeest occurred in Zambia [28]. This was mainly due to considerable numbers (23 500 in 2004) surviving in the Liuwa Plain National Park. According to Estes and East, this population might be one of the last large migratory populations that still follows historical wet- and dry-season migration routes into Angola. In 2004, around 25 500 wildebeest occurred in Zambia [27].

## Blue wildebeest numbers in the KAZA TFCA between 1925 and 2004

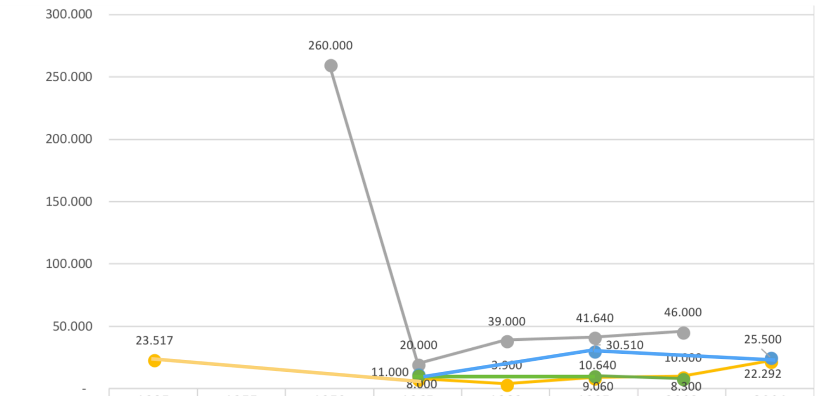


| | 1925 | 1955 | 1959 | 1965 | 1989 | 1995 | 2002 | 2004 |
|---|---|---|---|---|---|---|---|---|
| Angola | | | | 10.000 | | | | |
| Botswana | | | 260.000 | 20.000 | 39.000 | 41.640 | 46.000 | |
| Namibia | 23.517 | | | 8.000 | 3.900 | 9.060 | 10.000 | 22.292 |
| Zambia | | | | 12.360 | | 30.510 | | 25.500 |
| Zimbabwe | | | | 11.000 | | 10.640 | 8.300 | |

*Plains Zebra (Equus quagga)*

Estimates suggest a population of 29 314 plains zebras in Namibia in 1925/26 [24]. In 1975, around 16 000 zebras occurred in then South West Africa, of which 90% were in the Etosha National Park [29]. By 1992, the population had decreased to 9300 head [30]. The population in the ENP had decreased by 75% between 1960 and 1985 and has been stable or slightly declining ever since. The population on private land has tripled since 1972 [30]. By 2004, zebra populations had increased to 25 421 [30] and an estimated 40 000 in 2015 [30]. The trend has been different for the other KAZA countries. In 1992, Botswana still had a relatively large population of zebras (47 000) [30]. The population in the north-east along the Zimbabwe border has been shrinking by 40% every year since 1990 [31]. In 1997, the number had declined to 34 300 [32] but remained relatively stable until 2002 (34 294). In 2015, however, the zebra population had decreased to 9593 head [20]. Similar trends can be observed for Zambia and Zimbabwe. For both countries the populations increased and seemed to stabilise between 1992 and 2002, but strongly decreased in the next 13 years [30]. The zebra population in Angola is almost extinct [30].

## Plains zebra numbers in the KAZA TFCA between 1926 and 2016

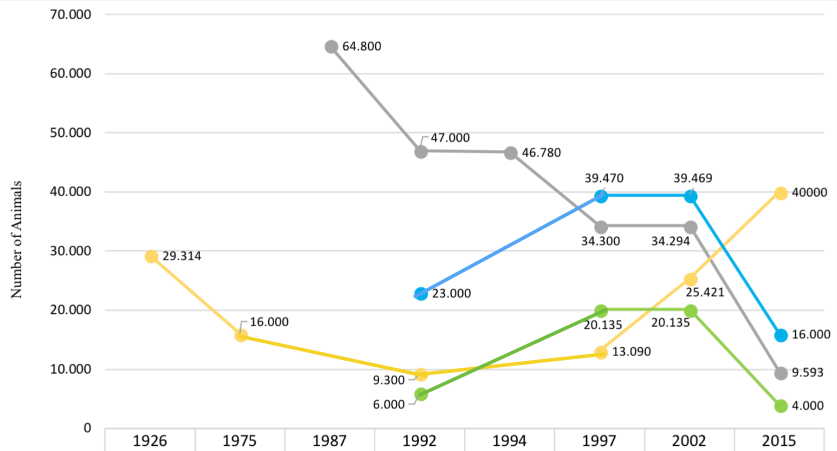


| | 1926 | 1975 | 1987 | 1992 | 1994 | 1997 | 2002 | 2015 |
|---|---|---|---|---|---|---|---|---|
| Botswana | | | 64.800 | 47.000 | 46.780 | 34.300 | 34.294 | 9.593 |
| Namibia | 29.314 | 16.000 | | 9.300 | | 13.090 | 25.421 | 40000 |
| Zambia | | | | 23.000 | | 39.470 | 39.469 | 16.000 |
| Zimbabwe | | | | 6.000 | | 20.135 | 20.135 | 4.000 |

*African Buffalo (Syncerus caffer)*

Buffalo numbers in Namibia have been relatively stable over time. According to Winterbach [33], around 600 buffalo occurred in Namibia in 1987. At the same time, Botswana had a population of 72 290 and Zimbabwe 66 590. While the populations in Namibia (1088) and Zimbabwe (80 000) had increased between 1987 and 1991, the Botswana population (41 380) was declining [33]. By 1995, the populations in both Zimbabwe (48 201) and Botswana (29 367) were declining while the Namibian population (2840) was still increasing [32]. The buffalo population of Namibia decreased in 1998, although numbers increased considerably in eastern present-day Zambezi region. Populations in Botswana (26 890) and Zimbabwe (50 330) seemed to stabilise [28]. In 2004, the population in Namibia's Caprivi Strip was between 1000 and 3000. The Zimbabwean population decreased (13 356) while the Botswana population increased to 82 674 [34].

**African buffalo numbers in the KAZA TFCA between 1975 and 2004**

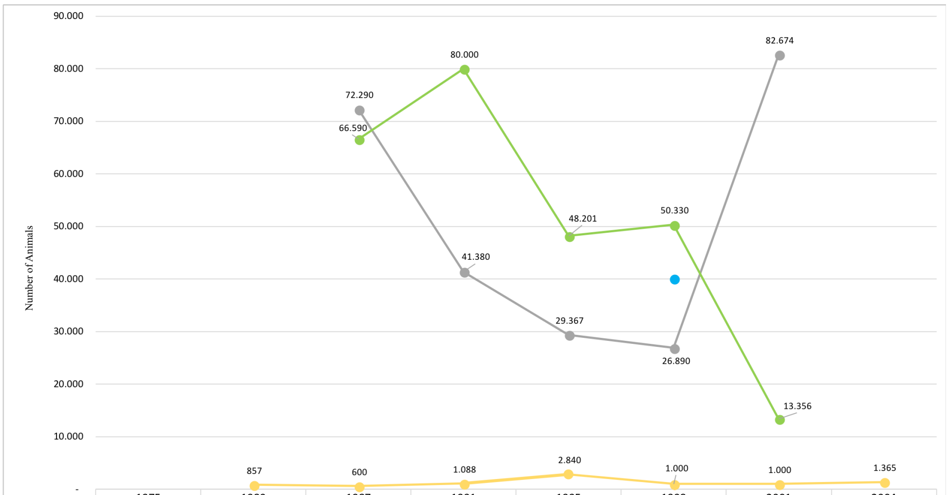


| | 1975 | 1980 | 1987 | 1991 | 1995 | 1998 | 2001 | 2004 |
|---|---|---|---|---|---|---|---|---|
| Angola | | | | | | 500 | | |
| Botswana | | | 72.290 | 41.380 | 29.367 | 26.890 | 82.674 | |
| Zambia | | | | | | 40.090 | | |
| Zimbabwe | | | 66.590 | 80.000 | 48.201 | 50.330 | 13.356 | |
| Namibia | | 857 | 600 | 1.088 | 2.840 | 1.000 | 1.000 | 1.365 |

*Supplementary Material 7*

**RECONSTRUCTION OF HISTORICAL OCCURRENCE OF SELECTED SPECIES**

*African Elephant (Loxodonta africana)*

Comparing the occurrence of the African elephant in 1934 (Shortridge), 1975 (Joubert & Mostert) and the most recent range identified by the Atlas of Mammals (Figure 4), the range increased between 1934 and 1975 and considerably declined thereafter. According to Shortridge, in 1934 elephants occurred in the Caprivi (today's Zambezi region) and Kaokoveld regions of then South West Africa (SWA) and occasionally migrated into north-west and north-east Ovamboland, the Outjo district and Okavango region. Although elephants mainly occurred in the northern regions of SWA, they migrated as far south as the Ugab river during the rainy season. Before the rainy season, elephants crossed the Okavango river from south-east Angola into SWA. Another "elephant region" was near the Khaudom/Shadum along the Omurambas that flow into the Okavango and Tshodilo Hills [Shortridge, 1934].

By 1975, the distribution range of elephants within the borders of today`s Namibia had clearly increased. According to Joubert and Mostert, small herds moved down the Hoanib drainage system into the western part of the Etosha National Park (ENP) and herds moved into Ovamboland from central ENP. Some herds moved from the Otjovansandu and Onaiso regions towards north-west Ovamboland and into the Kaokoland. Elephants moved from Damaraland into Angola and western present-day Zambezi region. The ENP had the highest concentrations of elephants in 1975. These herds moved eastwards along drainage lines coalescing into the Okavango river [Joubert & Mostert, 1975].

The current range is considerably smaller and resembles the occurrence pattern in 1934. The Atlas of Mammals data suggests that large numbers occur in the Zambezi region, Khaudom National Park and Etosha National Park. The Zambezi region is an important corridor for elephants migrating into the other KAZA countries [IUCN Elephant Specialist Group, 2008]. Elephants occur in southern Angola, but there is a clear cut along the southern border to Namibia [IUCN Elephant Specialist Group 2008]. In Botswana, elephants are concentrated in the northern regions. Their range covers approximately 100 000 $km^2$ and has been expanding over the last years. The elephants in Botswana are part of a larger population that stretches into Zimbabwe and north into Namibia's Zambezi region. The population possibly moves into Zambia and into Angola. Due to the expansion of elephant range in Botswana elephants move westwards into Khaudom and Nyae Nyae in Namibia [IUCN Elephant Specialist Group, 2008].

*Lion (Panthera leo)*

The pattern of lion distribution has also changed considerably since 1934. Shortridge recalls that lions mainly occurred in the north-west, north-east and eastern regions of SWA. A few lions existed in the Namib mountainous valleys as far south as the Kuiseb river. According to him, below 25ºS (latitude) lions had almost completely disappeared. Some lions followed prey along the Auob river to the south-east close to Upington. In the former Damaraland, they were sometimes found in the coastal regions from the Swakop and lower Kuiseb rivers in the south, around the Spitzkoppe to north of the Ugab river. Lions were widely distributed northwards of the Ugab throughout the Kaokoveld. The highest densities occurred around the Cunene Valley and some northern native villages. Sometimes, although rarely, they were also found close to Windhoek. Lions were found all over Ovamboland and were very common in the Okavango region. The present-day Zambezi region also had considerable lion populations that were evenly distributed and especially plentiful in the Kukweveld in central Zambezi [Shortridge, 1934].

The range in 1975 was similar to the range in 1934. According to Joubert and Mostert, lions were absent from central Namibia and had been eradicated from most farmland, Ovamboland and the Kaokoland plateau. Some lions remained in north-west Damaraland - formerly part of the Etosha National Park (ENP) - and in Kaokoland. Furthermore, they could still be found in the ENP and southern Kavango along the Botswana border. Some prides also persisted further south in Bushman- and Hereroland [Joubert & Mostert, 1975].

Today the range is considerably smaller. The Atlas of Carnivores recorded that lions have disappeared from most of the extreme north, southern and central regions of the country. Regions that have been identified as Lion Conservation Units (LCUs) in Namibia are the Kunene–Etosha population (increasing) and the Khaudom–Caprivi population (stable) [IUCN, 2006].

*Blue Wildebeest (Connochaetes taurinus)*

In 1934, based on the information collected by Shortridge, the blue wildebeest was the most widely distributed and most numerous antelope in South West Africa (SWA). High numbers in Ovamboland were constantly reinforced by migrations from the Etosha pan area in the rainy season, when water was available in pans and vleis. Their western limit was approximately the Kaokoveld–Ovambo border and only a few were reported from north-eastern Kaokoveld [35]. Wildebeest populations in the western Okavango and Grootfontein district used to migrate into the Kalahari in Botswana during the rains. Estes & East [2009] even suggest that most wildebeest in the region were part of a single population. In the dry season, these wildebeest concentrated around the Makarikari Pans, Etosha Pan, Lake Ngami, Chobe river, Okavango swamp, Ovamboland, the present-day Zambezi region, the Cubango and Cunene rivers in Angola as well as the Limpopo river in Botswana and South Africa [Estes & East, 2009]. Large numbers could also be found in the present-day Zambezi region [Martin, 2004]. West of the Waterberg in the Otjiwarongo district, numbers started decreasing and they were a rare sight in the Otjiwarongo and Okahandja districts. According to Shortridge, wildebeest were more plentiful in the Gobabis and Gibeon district, where they were abundant in the sand dunes east of the Nossob. The southern limit in SWA was the north-east of the Aroab district. The greatest concentrations occurred between the Okavango and Kwando rivers [Shortridge, 1934].

Distribution range collapsed considerably between 1934 and 1975. Based on the findings of Joubert and Mostert, wildebeest were restricted to the eastern part of the ENP, where they were also the most abundant. Very few still existed in the western portion of the park. They had disappeared from Kaokoland. To the east a few individuals could still be found on farms in the Gobabis and Grootfontein districts. They still occurred in northern Kavango and along the Namibia–Botswana border in the Kavango region. Some remained in Bushman- and Hereroland [Joubert & Mostert, 1975].

Today, the blue wildebeest has a smaller range than in 1934. Wildebeest are still present in ENP and in some other protected areas, for example the Khaudom National Park. Low densities can be found in communal areas. Some farms have started to gradually reintroduce the blue wildebeest. They have also been reintroduced to farms outside their natural historical range in the southern and western regions of the country [East, 1998].

*Plains Zebra (Equus quagga)*

Changes in the occurrence pattern of the plains zebra are similar to those of the blue wildebeest. In 1934, the plains zebra could be found in northern Damaraland, the northern and eastern Kaokoveld, Etosha Pan Area, Ovamboland as well as in the Okavango and present-day Zambezi regions. The herds in the southern Kaokoveld and in the northern and eastern parts of the Outjo District migrated into the Namutoni Game Reserve when water became scarce and concentrated around the Etosha pan. In the extreme north they also migrated westwards to the coast following the Cunene river. They occurred in Ovamboland as long as water was available in vleis and pans and mostly originated from the Namutoni Game Reserve. According to Shortridge, they sometimes wandered from the Etosha pan into the Otjiwarongo district. Some animals existed in northern Bechuananland. They became more common and plentiful between the western Caprivi (today's Zambezi Region) and Lake Ngami. They dispersed along the Omuramba-Omatako until Namukaub. Furthermore, they could be found in the Kungveld along the Khaudum, Shadum and Okavango rivers and flood area. They were numerous in the present-day Zambezi region [Shortridge, 1935].

By 1975, the range was considerably smaller. According to Joubert and Mostert, plains zebra were most densely populated in the eastern part of the ENP. From there they migrated to the north-east during the dry season along the Etosha pan. Herds from the south-eastern side of the pan moved towards the Andoni plain north of Namutoni and herds from the western side of the pan moved into southern Ovamboland. They did not occur in the centre of the ENP. In Kaokoland they were prevalent within the plateau regions. In the northern Kaokoveld they occurred from the escarpment transition zone down to the sub-desert Namib plains. The eastern border seems to be along the border with Botswana. They were absent from western Caprivi (present-day Zambezi region) and the extreme north [Joubert & Mostert, 1975].

Today, the plains zebra has a larger range than in 1934. According to the Atlas of Mammals, a large population still exists in the Etosha National Park. They can further be found on communal land and private farms in the North. Large populations prevail in northern Botswana, dispersing around protected areas [Hack et al., 2002].

*African Buffalo (Syncerus caffer)*

The pattern of occurrence of the African buffalo has declined considerably since 1934. In 1934, Shortridge confirmed that buffalo were common on the Angolan side of the Okavango river and along the lower reaches of the Kwando and Okavango rivers. In Namibia, they occurred in eastern and central present-day Zambezi region near the Mashi river, Hukweveld and the rivers around the Linyanti swamp. In the lower reaches of the Okavango they were found on both sides but were more plentiful in the eastern part. They were rare in the lower Okavango swamps but increased in numbers towards the Zambezi–Chobe watershed. Occasionally migrants from Angola could be found in the north-west of the Grootfontein district and in north-eastern Ovamboland [Shortridge, 1934].

By 1975, buffalo no longer occurred in Ovamboland. According to Joubert and Mostert, they were still present in the north-west of the Kavango region and along the Botswana border to the north-east. Very small numbers were reported for then Bushmanland south of the Aha mountains. However, Joubert and Mostert mentioned that they only occurred in very low numbers throughout. A herd of around 500 non-resident buffalos was sighted on the west bank of the Kwando and seemed to migrate from Botswana into SWA [Joubert & Mostert, 1975].

Today, buffalo only occur in the north-east, in particular in the Nkasa Rupara National Park (formely Mamili National Park). According to Winterbach [1996], smaller sub-populations occur in the Bwabwata National Park (formerly West

Caprivi Game Reserve) and the eastern part of then Bushmanland. A small group has been introduced to the Waterberg Plateau Park. The range in southern and western Botswana has been severely reduced and is now restricted to the northern areas [East, 1998]. They moved into Namibia and Zimbabwe [Winterbach, 1996]. Large numbers are found in the southern and central Okavango Delta. Smaller populations occur in the Chobe National Park and along the border with Zimbabwe in the north-east [East, 1998].

*REFERENCES OCCURENCE*

Atlasing of Namibia. Atlas of Mammals and Carnivores, Environmental Information Service. http://www.the-eis.com/atlas/ (2020).

East, R. & IUCN/SSC Antelope Specialist Group. African Antelope Database 1998. *Occasional Paper of the IUCN Species Survival Commission No.21* (1998).

Estes, R.D. & East, R. Status of the Wildebeest (Connochaetes taurinus) in the wild 1967 – 2005. *Wildlife Conservation Society Working Paper No.37* (2009).

IUCN. Regional Conservation Strategy for the Lion Panthera Leo in eastern and southern Africa. IUCN SSC Cat Specialist Group (2006).

IUCN/SSC African Elephant Specialist Group. Loxodonta Africana. The IUCN Red List of Threatened Species. Version 2017-3. http://www.iucnredlist.org (2008).

Joubert E & Mostert P.K.N. Distribution patterns and status of some mammals in South West Africa. Division of Nature Conservation and Tourism South West Africa Administration. Windhoek Scientific Society (1975).

Martin, R.B. Transboundary Species Project: the Southern Savannah Buffalo. Species Report for the Southern Savannah Buffalo in Support of the Transboundary Mammal Project of the Ministry of Environment and Tourism Namibia facilitated by the Namibia Nature Foundation and the Living in a Finite Environment (LIFE) Programme (2004).

Shortridge, G.C. The Mammals of South West Africa - A Biological Account of the Forms occurring in that Region. Volume 1 and 2. Namibia Scientific Society (William Heinemann Ltd., 1934).

Winterbach, H.E.K. The status and distribution of Cape buffalo Syncerus caffer caffer. *African Journal of Wildlife Research 1998* ***28 (3)*** (1996).
